# p53 Gene Targeting by Homologous Recombination in Fish ES Cells

**DOI:** 10.1371/journal.pone.0059400

**Published:** 2013-03-19

**Authors:** Yan Yan, Ni Hong, Tiansheng Chen, Mingyou Li, Tiansu Wang, Guijun Guan, Yongkang Qiao, Songlin Chen, Manfred Schartl, Chang-Ming Li, Yunhan Hong

**Affiliations:** 1 Department of Biological Sciences, National University of Singapore, Singapore, Singapore; 2 Department of Bioresource, National Institute for Basic Biology, Okazaki, Japan; 3 Yellow Sea Fisheries Research Institute, Chinese Academy of Fisheries Sciences, Qingdao, China; 4 Physiological Chemistry, Biocenter of the University of Würzburg, Würzburg, Germany; 5 Institue for Clean Energy & Advanced Materials, Southwest University, Chongqing, China; 6 Division of Bioengineering, School of Chemical and Biomedical Engineering, Nanyang Technological University, Singapore, Singapore; University of Frankfurt - University Hospital Frankfurt, Germany

## Abstract

**Background:**

Gene targeting (GT) provides a powerful tool for the generation of precise genetic alterations in embryonic stem (ES) cells to elucidate gene function and create animal models for human diseases. This technology has, however, been limited to mouse and rat. We have previously established ES cell lines and procedures for gene transfer and selection for homologous recombination (HR) events in the fish medaka (*Oryzias latipes*).

**Methodology and Principal Findings:**

Here we report HR-mediated GT in this organism. We designed a GT vector to disrupt the tumor suppressor gene *p53* (also known as *tp53*). We show that all the three medaka ES cell lines, MES1∼MES3, are highly proficient for HR, as they produced detectable HR without drug selection. Furthermore, the positive-negative selection (PNS) procedure enhanced HR by ∼12 folds. Out of 39 PNS-resistant colonies analyzed, 19 (48.7%) were positive for GT by PCR genotyping. When 11 of the PCR-positive colonies were further analyzed, 6 (54.5%) were found to be *bona fide* homologous recombinants by Southern blot analysis, sequencing and fluorescent *in situ* hybridization. This produces a high efficiency of up to 26.6% for *p53* GT under PNS conditions. We show that *p53* disruption and long-term propagation under drug selection conditions do not compromise the pluripotency, as *p53*-targeted ES cells retained stable growth, undifferentiated phenotype, pluripotency gene expression profile and differentiation potential *in vitro* and *in vivo*.

**Conclusions:**

Our results demonstrate that medaka ES cells are proficient for HR-mediated GT, offering a first model organism of lower vertebrates towards the development of full ES cell-based GT technology.

## Introduction

Gene targeting (GT) in mouse embryonic stem (ES) cells has been used as a powerful tool for analyzing gene function [1]. In this approach, precise alterations are introduced into ES cells at particular loci by gene replacement, and targeted ES cells are introduced into early embryos for the formation of chimeras in which transplanted ES cells contribute to many lineages including the germline. Crossing of germline chimeras lead to the production of animals that are heterozygous or homozygous for the targeted locus. GT in ES cells has been reported also in human [2] and rat [3]. Because of the availability of pluripotent ES cell lines and the possibility of germline chimera formation, however, the production of knockout animals from targeted ES cells has long been limited to mouse [4] before its recent expansion into rat [5].

The effort towards GT has steadily been attempted in non-mammalian species such as lower vertebrates, in particular zebrafish and medaka (*Oryzias latipes*). In zebrafish, Collodi and his colleagues have reported targeted insertion of a plasmid by HR in zebrafish ES cell cultures [6]. More importantly, zebrafish with targeted gene mutations have been generated by using zinc finger nucleases (ZFNs) [7], [8] and transcription activator-like effector nucleases (TALENs) [9].

We and others use the medaka (*Oryzias latipes*) as a model organism to develop the GT technology. Like zebrafish, this fish is an excellent model for analyzing vertebrate development [10]. In this organism, we have obtained various stem cell lines of diploid ES cells [11], haploid ES cells [12], [13] and adult germ cells [14], [15], and established procedures for gene transfer *in vitro* [16], [17], [18], [19] and *in vivo* [17], [19], [20], positive-negative selection (PNS) to enrich for HR-mediated GT in ES cells [16], and chimera formation [21].

In order to fully develop the GT technology in medaka, we chose the *p53* gene as a model. *p53* is best known as the “guardian of the genome” and tumor suppressor, as *p53* mutations occur in 50% of human cancers [22]. *p53* is highly conserved across animal phyla, as its mutation increases the incidence of tumor formation in mouse [22] and fish [23], [24]. In mice, *p53* is involved in several other important processes such as senescence and ageing [25]. *p53* has been targeted by HR in ES cells of mouse [26] and rat [5]. Previously, we have shown the lack of ultraviolet-light inducibility of the medaka *p53* gene [27], and reported a first attempt toward the development of GT in medaka ES cells, in which p53 gene was cloned for the construction of GT vector pGTp53 [28]. In this work, the genuine GT event was not described, thus parameters for PNS and GT efficiency remained speculative. We made use of the pGTp53 vector [28], to continue the effort towards the establishment of GT technology in fish ES cells. pGTp53 was devised to disrupt the medaka *p53* by HR on the basis of PNS to allow for enrichment for HR events [29].

This study was aimed at continuing our effort for improving procedures and efficiency for HR-mediated true *p53* GT in medaka ES cell lines. We show the effectiveness of PNS procedure in enrichment for the HR event and a high efficiency of GT in medaka ES cells. More importantly, we demonstrate the retention of pluripotency of medaka ES cells after long-term drug selection and targeted *p53* disruption.

## Results

### Gene Transfer and Selection Strategy

**Figure 1:**
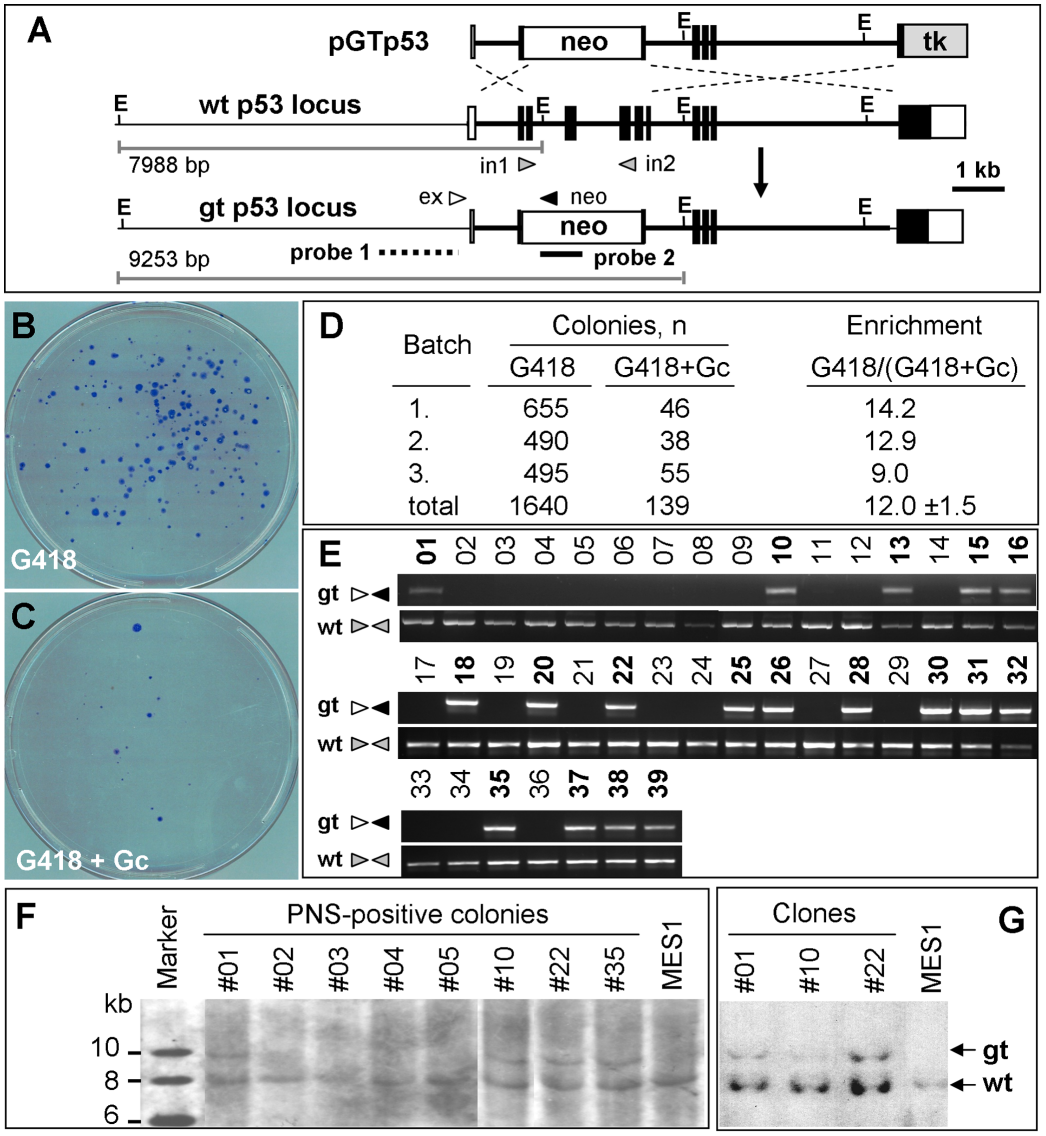
p53 gene targeting in MES1. (**A**) Targeting vector pGTp53. gt, gene-targeted; wt, wildtype; filled box, translated exon; open box, untranslated exon; thin line between boxes, intron; thick line, region included in pGTp53; *neo*, STneo cassette; *tk*, STk cassette; E, *EcoR* I site. Arrowheads depict the position and extension direction of primers (in1 and in2, *p53* primers whose sequences are absent in pGTp53 and the targeted locus; ex, external primer; *neo*, *neo* primer). Probes and *EcoR* I-digests of predicted sizes for Southern analysis are indicated. (**B** and **C**) Colony formation. Colonies formed from pGTp53-transfected MES1 cells in the presence of G418 alone (B) and G418 plus Gc (C) were Giemsa-stained at day 23 of clonal growth. (**D**) PNS enrichment factor. (**E**) PCR detection of the GT event. Genomic DNA was isolated from 39 PNS-resistant colonies and subjected to PCR detection by using primers (arrows; for positions see Figure 1A) specific to the targeted (gt) and wildtype (wt) locus. Numbers above lanes, PNS-resistant colonies. Positive colonies are shown in bold. (**F**) Southern analysis of representative colonies. (**G**) Southern analysis of representative clones from colonies after ≥20 passages of propagation.

For HR-mediated GT experiments, we made use of the GT vector pGTp53 (Figure 1A) that has been described previously [28]. Gene transfer of linearized pGTp53 vector into medaka ES cells was performed by using the GeneJuice reagent (Novagen) [14], [16], [18] in 6-well plates. Each well was considered as a pool for screening of HR-events after gene transfer. Upon HR, the *neo* would be co-integrated, while the *tk* would be recombined away, with the homologous recombinant being resistant to G418 and gancyclovir (Gc) owing to the expression of *neo* and the absence of *tk*. Upon random integration (RI), both the *neo* and *tk* would be co-integrated, with random integrants being resistant to G418 but sensitive to Gc owing to the expression of both *neo* and *tk*. Therefore, the PNS by using G418 and Gc is predicted to enrich for HR events by eliminating RI events via their sensitivity to Gc and non-transgenic cells via their sensitivity to G418 (Figure 1B and C).

### High HR Activity in different Medaka ES Cell Lines

There are three medaka ES cell lines, namely MES1-3 [11]. We first determined whether they possessed reasonable HR activity as a precondition for GT. To this end, the three cell lines were transfected in 12-well plates with pGTp53 and the putative HR event was detected at 5 days post transfection (dpt), by PCR analysis using primers specific to the targeted *p53* locus (Figure 1A). This led to the detection of a GT-specific PCR product in all of the six batches of pGTp53-transfected ES cells but not in mock-transfected cells (Figure S1). PNS by using G418 (500 µg/ml) plus Gc (5 µM) increased the yield of the GT-specific PCR product. Importantly, a single round of PCR produced an easily detectable band in MES1 and MES2 but a faint band in MES3 (Figure S1). The band for the targeted allele became more intense after a 2^nd^ round of PCR. Therefore, the three ES cells lines possess high but different levels of cellular HR activity, and MES1 was chosen for subsequent experiments because of its high HR activity and documented ability for chimera formation [21], [30] and retention of pluripotency after gene transfer and long-term drug selection [16].

### Efficient *p53* Gene Targeting

Compared to RI, HR is a rare event even in mouse ES cells [1]. Previously, we have shown the effectiveness of PNS in model systems of fish cell culture [28], [29]. We wanted to determine the efficiency of PNS for enrichment for the HR event in medaka ES cells. To this, 10^6^ transfectants were seeded in 10-cm dish and grown in the presence of G418 alone or together with Gc, and colony formation was examined after 23–28 days of culture. In a typical experiment, selection with G418 alone produced 100∼300 colonies per dish, while the number of colonies decreased to 5–25 per dish upon PNS (Figure 1B and C). A statistical analysis of three independent experiments led to an enrichment factor of 12.0±1.5 for PNS in pGTp53-transfected MES1 cells (Figure 1D), demonstrating the effectiveness of PNS for enriching for putative homologous recombinants in medaka ES cells.

We utilized four procedures to identify genuine homologous recombinants. A PCR-based genotyping procedure was first used to screen for the targeted *p53* locus, which was amenable for PCR amplification by using an external primer and *neo* primer (Figure 1A). From two batches of experiments, 39 colonies were successfully transferred and expanded, 19 (48.7%) were positive for PCR-genotyping (Figure 1E and Table S2 in File S1). When 11 of the PCR-positive colonies were then subjected to Southern blot analysis, 6 (54.5%) turned out to contain a 9.2-kb band and an 8-kb band without any visible additional bands, whereas normal MES1 cells has only the 8-kb band (Figure 1F and Table S2 in File S1). The 8-kb and 9.2-kb bands conformed to the predicted sizes of genomic *EcoR* I-digests for the wildtype allele (7988 bp) and targeted allele (9253 bp; Figure 1A; for detail refer to File S2). The targeted allele remained unchanged during ≥20 passages for expansion into individual clones (Figure 1G). Fluorescent *in situ* hybridization in one of the 6 clones (clone #22) demonstrated the presence of a single *neo* signal in the cells at different cell cycle phases (Figure S2). Sequencing revealed the predicted junction sequence for the targeted *p53* allele (Figure S3). Taken together, *p53* GT has efficiently occurred without detectable RI events and the targeted *p53* allele is as stable as the wildtype allele during clonal expansion.

### Retention of Pluripotency *in vitro*

**Figure 2:**
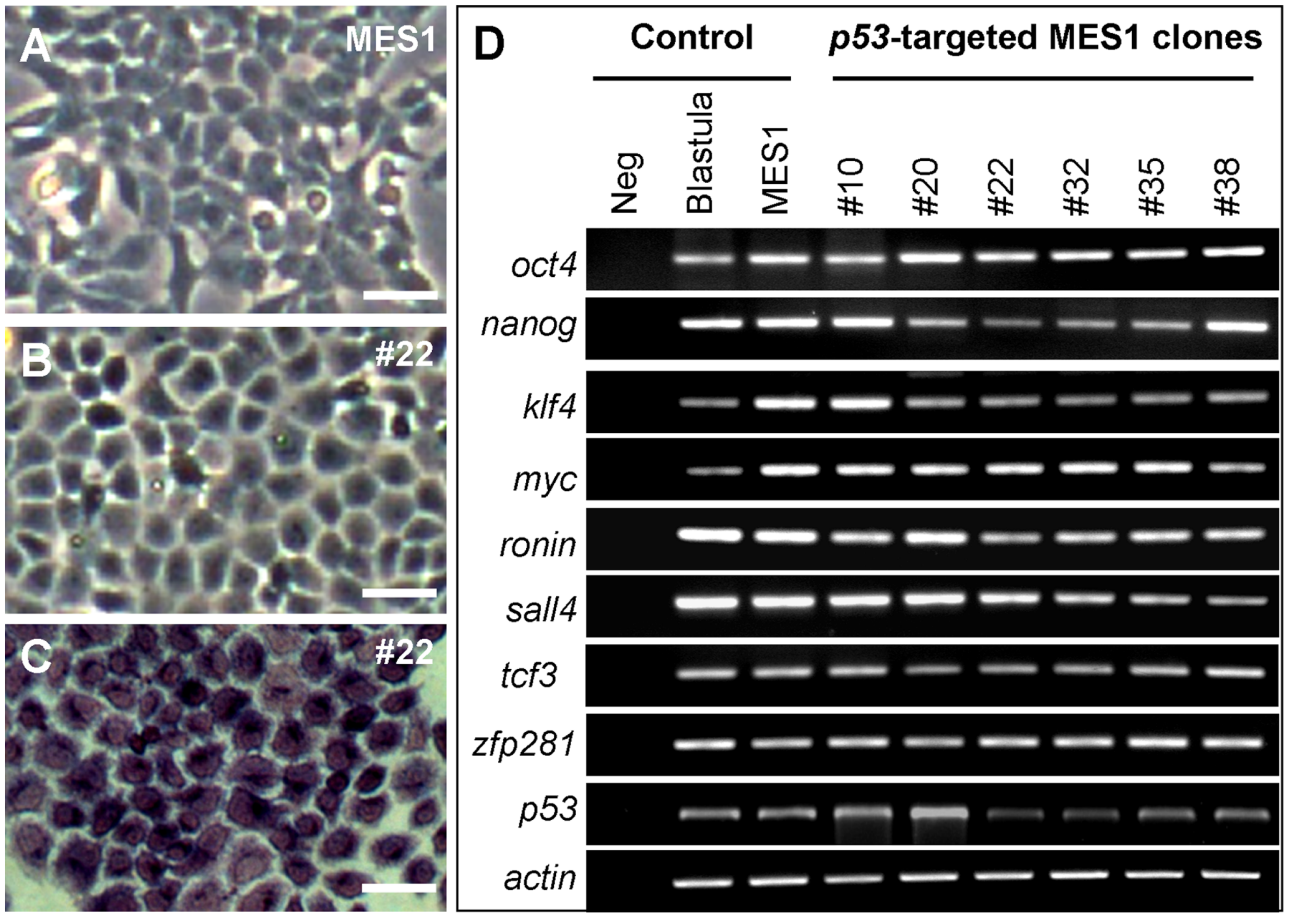
Pluripotency *in vitro*. (**A**) Phenotype of parental MES1 cells. (**B**) Phenotype of *p53*-targeted clone #22. (**C**) Alkaline staining of clone #22. (**D**) Pluripotency gene expression profile in five *p53*-targeted MES1 clones. neg, negative control without cDNA template. *actin* was used as a loading control. Blastula embryos and parental MES1 were used as positive controls. Scale bars, 25 µm.

To determine whether the pluripotency was compromised by drug selection and *p53* GT, normal MES1 and its *p53*-targeted counterparts including clone #22 were analyzed for differentiation potential *in vitro* and chimera competence *in vivo*. Under the conditions for undifferentiated growth, clone #22 exhibited the ES-cell phenotype such as a small size and round shape similar to that of MES1 (Figure 2A and B), and high alkaline phosphatase activity (Figure 2C), a general marker of fish stem cells [11], [12], [14], [17]. Moreover, all the six *p53*-targeted clones examined, like normal MES1 cells, did exhibit a high level of expression of 9 pluripotency genes including *nanog*, *oct4* and *klf4* (Figure 2D), which have recently been shown to be associated with an undifferentiated state of medaka stem cells in culture [12], [31]. As expected, the *p53* transcript was found at a reduced level in 4 out of 6 clones, namely clones 22, 32, 35 and 38, as compared to normal MES1 cells (Figure 2D), in consistence with heterozygosity at the *p53* locus in these clones.

**Figure 3:**
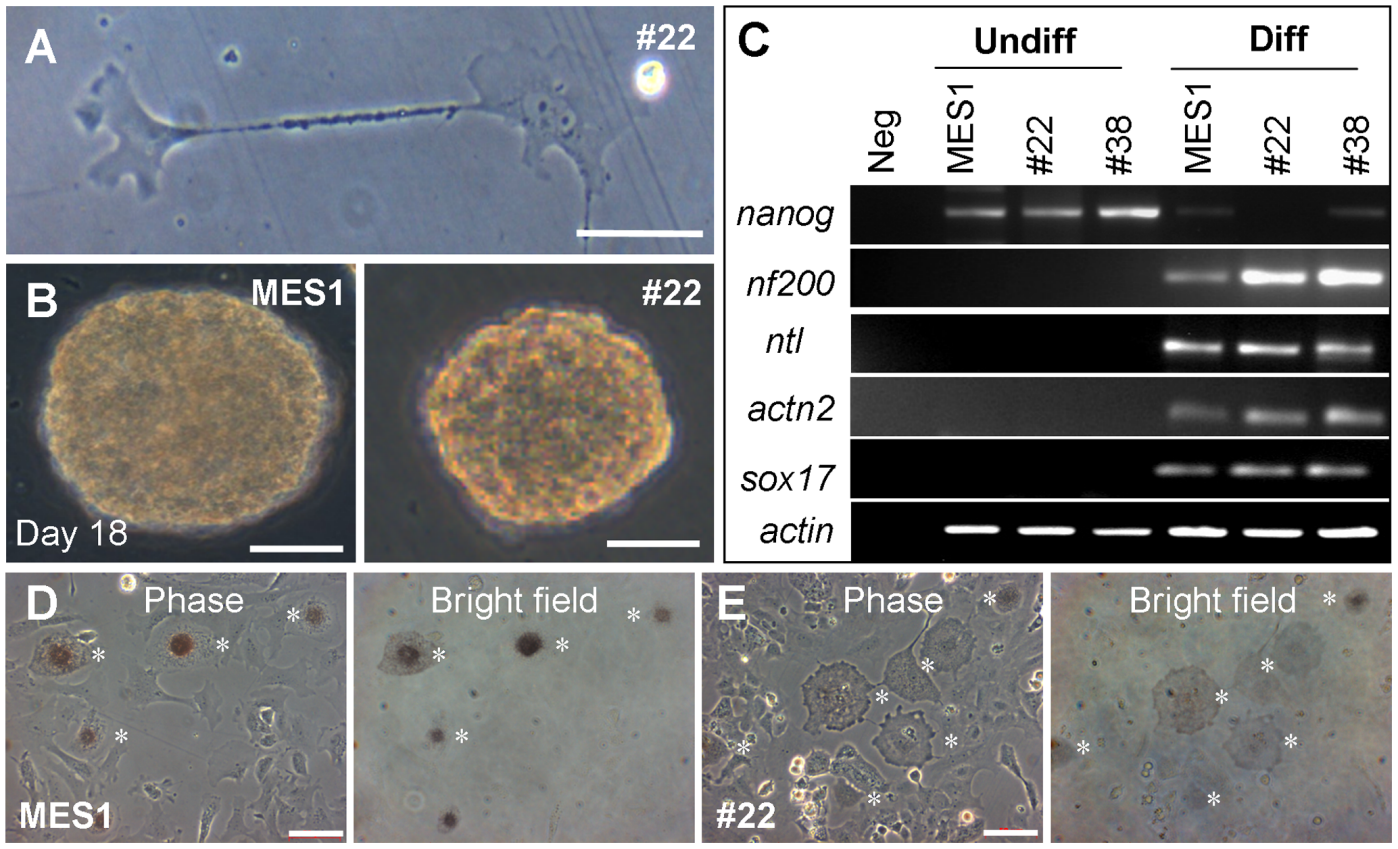
Differentiation *in vitro*. (**A**) Spontaneous differentiation of *p53*-targeted MES1 clone #22 into neurons. (**B**) Embryoid body (EB) formation of MES1 (left) and clone #22 (right). (**C**) Expression of differentiation genes upon induced differentiation by EB formation. *nf200*, neurofilament 200; *ntl*, no tail; *actn2*, actinin 2. (**D** and **E**) Directed differentiation into melanocytes (asterisks) by forced Mitf expression from pXmitf. Scale bars, 50 μm.

Under the conditions for differentiation induction [11], *p53*-targeted cells were able to differentiate spontaneously into various specialized cell types including neurons with long and thin cytoplasmic extensions (Figure 3A). They were capable of induced differentiation via embryoid body (EB) formation in suspension culture (Figure 3B), and they lost *nanog* expression and gained expression of several differentiation genes including the neuroectodermal marker *nf200*, the mesodermal markers *ntl* and *actinin2* as well as the endodermal marker *sox17* (Figure 3C), as have previously been shown for normal medaka ES cells [11], [12], [31], [32]. Normal MES1 cells can be directed for differentiation by forced Mitf (microphthalmia-associated transcription factor) expression into melanocytes [19], [33]. Upon transfection with pXmitf, vector expressing the *Xiphophorus* Mitf [19], the parental MES1 and *p53*-targeted counterparts produced pigmented melanocytes at similar efficiencies (Figure 3D and E; Figure S4). Taken together, medaka ES cells after long-term drug selection and *p53* GT retain the stem cell phenotype and pluripotency for spontaneous, induced and directed differentiation *in vitro*.

### Retention of Pluripotency *in vivo*

**Figure 4:**
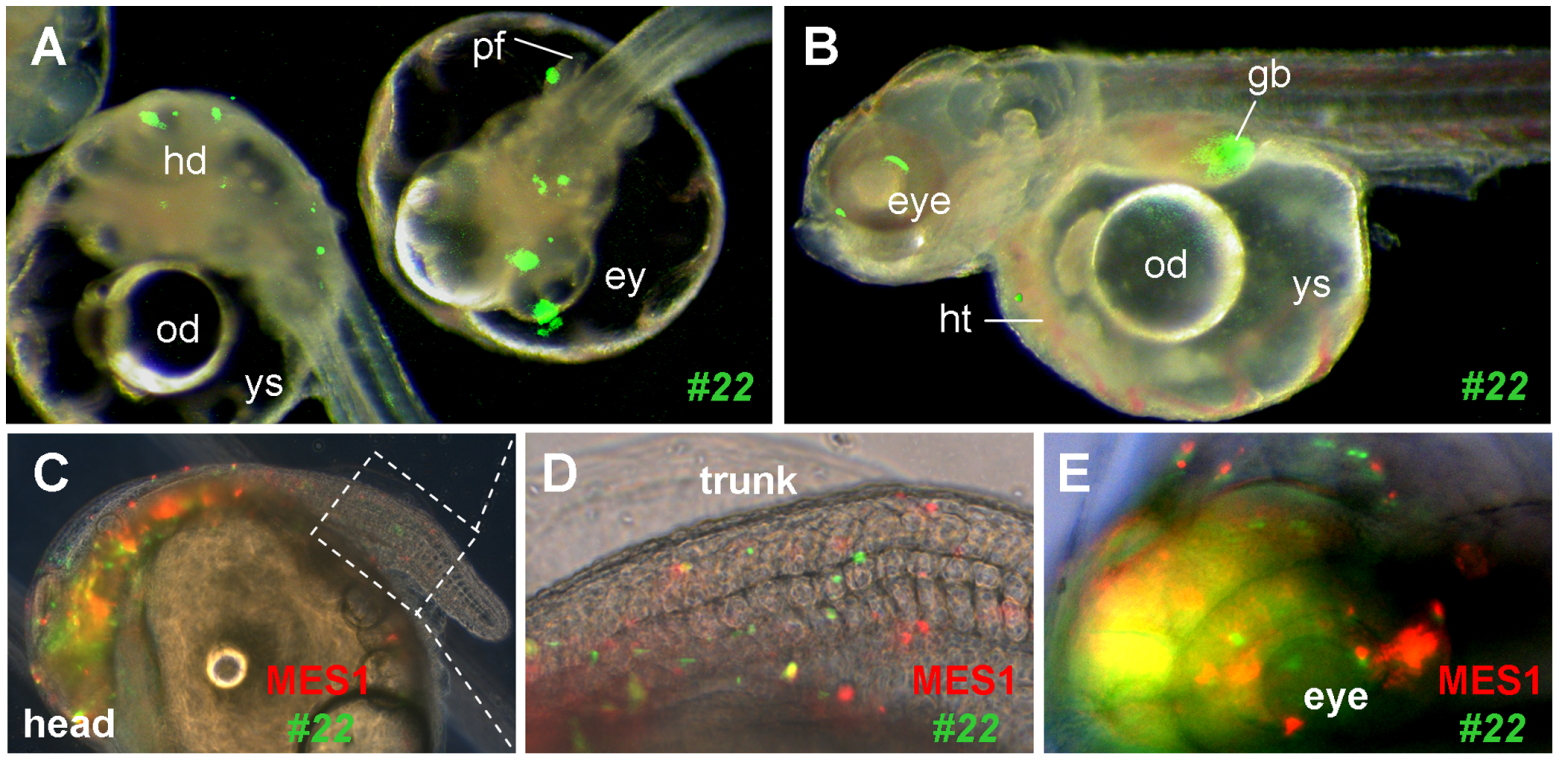
Pluripotency *in vivo*. Embryos at the midblastula stage were transplanted with ES cells and photographed at day 3 (**A**) and 7 post fertilization (**B–E**). (A and B) Merged micrographs of chimeras, showing GFP-labeled ES cells of *p53*-targeted clone #22 in many compartments and organ systems including the eye (ey), head (hd), heart (ht), gall bladder (gb) and pectoral fin (pf). od, oil droplet; ys, yolk sac. (C–E) Co-distribution of parental MES1 and *p53*-targeted clone #22 in the developing chimera at day 7 post fertilization. Following genetic labeling by plasmid transfection, parental MES1 (red) and clone #22 (green) were mixed at 1∶1 ratio and transplanted at approximately 200 cells per blastula. (**D** and **E**) Larger magnification of the posterior trunk framed in (C) and anterior end. The embryo is 1 mm in diameter.

We have previously shown that MES1 cells after stable gene transfer and drug selection were able to give rise to 100% chimera formation and that these donor cells contributed to a wide variety of tissues/organs during chimeric embryogenesis [12], [16], [21], [30], [34]. In order to test whether this property was retained after long-term drug selection plus *p53* GT, one of *p53*-targeted clones, clone #22, was genetically labeled with GFP via transfection by pCVpf and introduced into host blastulae. This generated 100% GFP-positive chimeras in which the GFP-positive donor cells were distributed to many compartments during early embryogenesis and to many tissues/organs at advanced stages (Figure 4A and B). Upon co-transplantation into blastula hosts, GFP-labeled cells of clone #22 were not different from RFP-labeled normal MES1 cells in distribution and differentiation in developing chimeric embryos (Figure 4C-E; Figure S5). Thus, *p53*-targeted ES cells have retained pluripotency *in vivo*.

**Figure 5:**
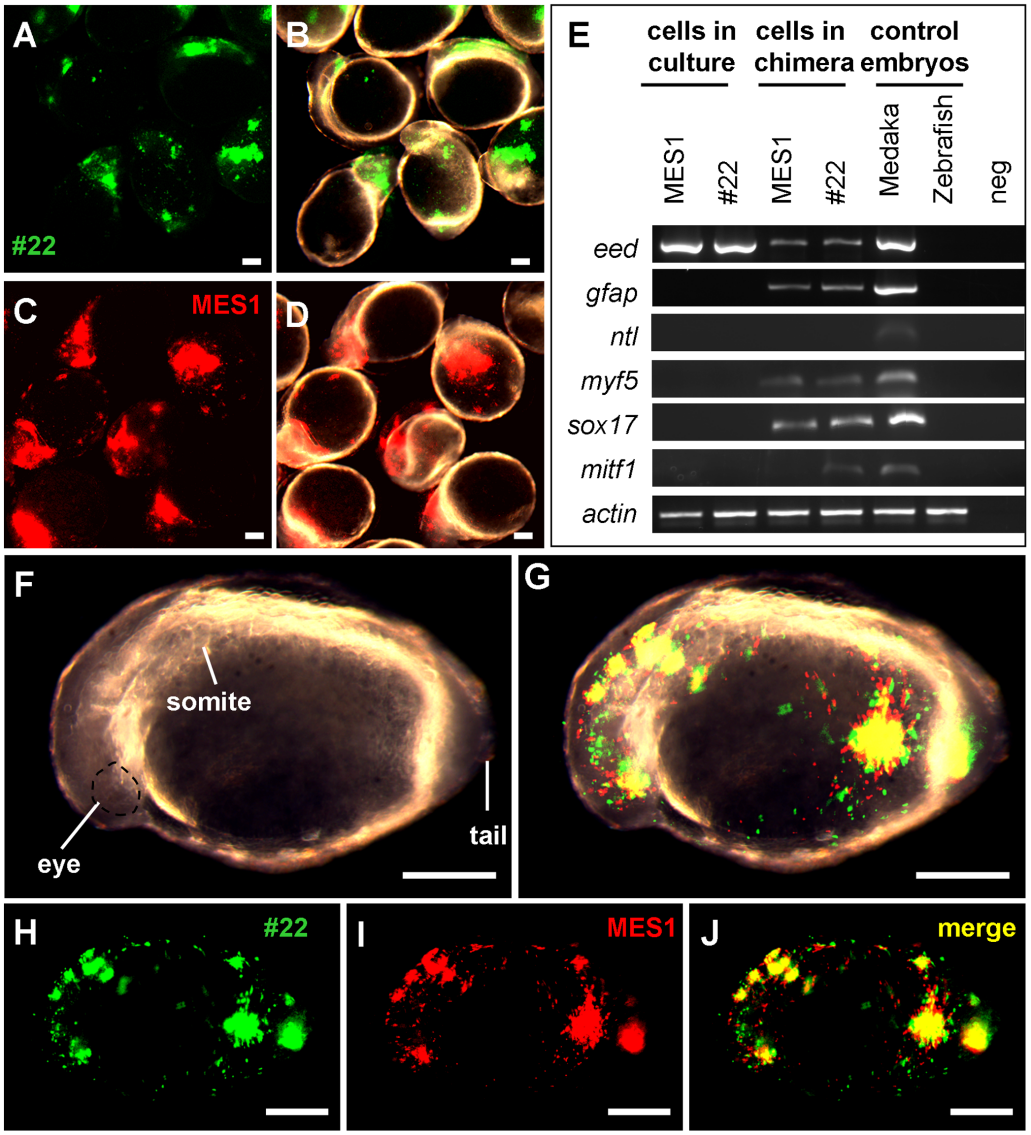
Chimeric assay of pluripotency *in vivo*. Zebrafish blastulae were used as the host for transplantation of MES1 (red), #22 (green) or both and analyzed at 1 dpf by microscopy and RT-PCR. (**A** and **B**) Chimeras by transplantation of #22 cells on fluorescent micrograph (A) and merge between fluorescent and brightfield optics (B). (**C** and **D**) Chimeras by transplantation of MES1 cells on fluorescent micrograph (C) and merge between fluorescent and brightfield optics (D). (**E**) RT-PCR analysis of gene expression. MES1, #22, 1-day-old embryos of medaka and zebrafish were used for comparison. Primers used were specific to medaka cDNAs, except for β-actin primers that amplify the β-actin cDNA of both medaka and zebrafish. (**F**–**J**) Chimera by cotransplantation of MES1 and #22 cells on brightfield (F), brightfield-fluorescent merge (G), GFP fluorescence (H), RFP fluorescence (I) and merge between GFP and RFP optics (J). Scale bars, 200 µm.

Recently, we have shown that interordinal chimera formation between medaka and zebrafish is a powerful tool for analyzing stem cell differentiation [35]. Specifically, interordinal chimera formation allows for a molecular analysis of ES cell differentiation along multiple cell lineages through RT-PCR assays by using primers that are specific to donor cell cDNAs. We adopted this approach. *p53*-targeted cells (clone #22) exhibited wide distribution to major compartments of 1-day-old zebrafish host embryos (Figure 5A and B). A similar observation was made also on parental MES1 cells (Figure 5C and D). The detection of medaka three germ layers (ectodermal, *eed* and *gfap*; mesodermal, *myf5*; endomermal, *sox17*) and neural crest (*mitf1*) markers in zebrafish host by RT-PCR revealed that *in vivo* differentiation potential of both parental MES1 cells and *p53*-targeted ES cells (#22 clone) (Figure 5E). Co-transplantation experiments revealed that MES1 and #22 cells had highly overlapping distribution (Figure 5F–J). Hence, we conclude that, as in mouse ES cells, drug selection and GT do not compromise the pluripotency of medaka ES cells.

## Discussion

Medaka and zebrafish are excellent twin model organisms for analyzing vertebrate development [10], studying stem cell biology [11], [12], [14], [17], [30], [36], [37] and more importantly, for developing the ES cell technology [16], [28], [29]. As a key step towards the full development of the ES cell technology in this organism, the present work has addressed five important issues. First, we show that all the three medaka ES cell lines possess a significant level of cellular HR activity, allowing for easy detection of HR events as early as just a few days of culture post transfection even in the absence of enrichment by drug selection. Second, we reveal that PNS is effective in enriching for HR events, producing a 12-fold enhancement factor, which is within the range of 2∼100 folds reported for mouse ES cells. Third, we demonstrate that *p53* GT in medaka ES cells occurs at a frequency 26% under PNS condition, a proficiency that is 13 times higher than a ∼2% efficiency for *p53* GT in ES cells of mouse [38] and rat [5]. With this high proficiency, it is possible to obtain a sufficient number of cell clones with a targeted locus from a few dozens of PNS-resistant colonies in a single transfection experiment. Fourth, our finding that all clones examined in this study are stable in growth and genetic stability indicate that a single copy of wildtype *p53* gene is sufficient for ES cell maintenance, in accordance with the observation that *p53* heterozygosity is sufficient to support ES cell maintenance in mouse [26] and rat [5] as well as normal development in medaka [24]. Finally and most importantly, we have provided compelling evidence that *p53*-targeted medaka ES cells retain pluripotency *in vitro* and *in vivo*, thus establishing medaka as first lower vertebrate organism to fully develop the HR-based GT technology.

Site-specific gene alterations can also be achieved by using engineered sequence-specific endonucleases such as ZFNs and TALENs. The ZFN or TALEN approach can introduce only minor additions or deletions in an unpredictable manner and are thus limited to gene disruption. Furthermore, this approach may also produce off-target alterations that are difficult to predict and detect. In contrast, HR-based GT generates precisely designed gene replacement, which allows for both gene disruption and correction and essentially alleviate the off-target concern. Therefore, the HR-based GT in ES cells followed by germline transmission still represents the approach of choice to engineer the genome at the best precision [1].

This study has focused on, successes in, HR-based GT in medaka ES cells by using *p53* as a model. This success corroborates and extends our previous effort with the same HR vector in MES1 cells [28], and extends the early study by demonstrating the first true success in *bona fide* GT in MES1 cells and the retention of pluripotency *in vitro* and *in vivo*. The next key step is to achieve germline transmission for whole animal production, as has been done in mouse [1] and rat [5]. In zebrafish, blastula cells after short-term culture can form germline chimeras [39]. In medaka, germline chimera formation from ES cell lines has not yet been described, in spite of continuous efforts [21], [30], [34]. Our previous study has indicated that medaka germ cell formation appears to be controlled by a cell-autonomous mechanism [40], raising the possibility that the medaka germline is inaccessible to colonization by cultured ES cells. Recently, we have generated medaka haploid ES cells capable of germline transmission by semicloning [12]. It is anticipated that GT in these haploid ES cells in combination with semicloning will provide an alternative approach for the production of knockout animals in this organism. The procedures and efficiencies established in present study will offer valuable information for GT in medaka haploid ES cells.

## Materials and Methods

### Fish and Reagents

This study was carried out in strict accordance with the recommendations in the Guide for the Care and Use of Laboratory Animals of the National Advisory Committee for Laboratory Animal Research in Singapore and approved by this committee (Permit Number: 27/09). Medaka strain *af* was maintained under an artificial photoperiod of 14-h light to 10-h darkness at 26∼28°C as described [19], [21]. Embryos were maintained at 26∼28°C and staged as described [41]. Unless otherwise indicated, chemicals were purchased from Sigma, enzymes and PCR reagents were from Promega and TaKaRa.

### Cell Culture

Maintenance of the medaka ES cell lines MES1-3 were maintained at 28°C under feeder-free culture conditions on gelatin-coated substrata in culture medium ESM4 and staining for alkaline phosphatase activity were performed as previously described [11], [42].

### Plasmids

The GT plasmid pGTp53 targeting the medaka *p53* gene was described [28]. Vectors pCVpf and pCVpr were used for drug selection and GFP or RFP expression for cell labeling [19]. Plasmid DNA was prepared by using the Qiagen Maxi and Midi preparation kits (Qiagen, Germany).

### Cell Transfection and Colony Isolation

Cell transfection with plasmid DNA by using the GeneJuice reagent (Novagen) and drug selection were performed as described [14], [16], [18]. Briefly, cells seeded in 6-well plates were incubated with linearized pGTp53 vector in a serum-free medium for 6 h. After medium change to ESM4, the cells were grown for 3 days and trypsinized into single cells. A quarter of the cells were collected for the PCR analysis (see below), and the remainder were subcultured into 10-cm gelatin-coated Petri dishes (plating) for PNS by using G418 (Gibco) at 500 µg/mL and Gc (Cymevan, Syntex Arzneimittel GmbH, Switzerland) at 5 µM.

After plating, pGTp53-transfected MES1 cells were grown under drug selection for up to 28 days. Dishes with ≤200 colonies were subjected to manual colony isolation. After transferring the PNS-resistant colonies into 96-well plate, a monolayer formed during 10–14 days of culture, with medium being changed every 3–5 days. After 7–12 days of culture, colonies were split into three 96-well plates. Two plates were used for by Southern (plate A) and PCR (plate B) analyses (see below), respectively, where the third was used as master plate (plate C) for cell propagation.

#### Micro-extraction of DNA

DNA samples from 96-well plates were isolated by using the micro-extraction procedure [43] with minor modifications. Briefly, the DNA was extracted by adding cell lysis buffer (10 mM Tris-HCl, pH7.5, 10 mM EDTA, 10 mM NaCl, 0.5% sarcosyl, and 500 µg/mL proteinase K), incubation at 55°C for 12–16 h and precipitation by NaCl and cold ethanol (1.5 µL of 5 M NaCl to 100 µL of absolute ethanol). After the 70% ethanol wash, 50 µL of autoclaved Milli-Q water was added to each well. DNA was dissolved and restored at 4°C until use.

### PCR

PCR-based genotyping was conducted from pooled cell populations or single PNS-resistant colonies by two sequential rounds of PCR. The first round of PCR was run for 35 cycles (94°C for 30 s, 60°C for 30 s and 72°C for 90 s) in a 25-µL volume containing 2 µl of DNA prepared above. The second round of PCR was similarly run for 30 cycles in a 25-µL volume containing 2 µL of the 10 times diluted 1^st^ round PCR product. PCR primers are listed in Table S1 in File S1. The PCR screening of PNS-resistant clones was performed using DNA samples prepared from 96-well plate. PCR products were separated on a 1.0% agarose gel and documented with a bio-imaging system (Synoptics, Cambridge).

For RT-PCR, total RNA was isolated by using the Trizol Reagent (Invitrogen). Synthesis of cDNA templates was primed with oligo (dT)_18_ by using M-MLV transcriptase (Invitrogen). The cDNA reaction was diluted with water to 10 ng/µL. RT-PCR was run as previously described [12], [14]. PCR primers are listed in Table S1 in File S1.

### Southern Analysis

DNA extraction was similarly done as described above but to a larger scale. Ten µg of genomic DNA was digested with *EcoR* I enzyme at 37°C for 30–40 h. The digested DNA fragments were separated on a 0.8% TBE agarose gel by electrophoresis at 30 V for 16 h at 4°C. After photograph and de-purination treatment with 0.2 M HCl for 10 min, the DNA was transferred to Hybond N+ nylon membrane (GE healthcare, USA) for 5 h by alkaline buffer (0.5 M NaOH, 1.5 M NaCl). Following transfer, the membrane was pre-hybridized with DIG-Easy solution (Roche) containing 100 µg/ml of pre-boiled calf thymus DNA fragments (≤2 kb in size) at 42°C of 12–16 h. The probe was labeled with digoxygenin (a mixture of 100∼1000-bp fragments synthesized from a 1.5-kb 5′-external sequence; for detail see Table S1 in File S1) by random-primed DNA synthesis (Roche), then hybridized to the membrane at 42°C in DIG-easy solution containing 100 µg/ml of calf thymus DNA for 40 h. Non-specific binding was removed by washing in 2 × SSC/0.5% SDS (twice), and 0.1 × SSC/0.5% SDS at 65°C for 10 min each. Followed by an overnight incubation at 4°C with a 1∶5000 diluted anti-DIG-POD antibody (Roche) in 1 × maleic acid solution containing 1.5% blocking reagent (Roche), the hybridization signal was visualized after chemiluminescent reaction by using the ECL reagent (Amersham Biosciences) and developed by exposure for 8 h to films (Kodak).

### Sequencing

DNA fragments were subcloned into pGEM-T easy vector (Promega) and sequenced on an ABI3100 automatic sequencer (Applied Biosystems). Sequence analyses were performed by using the Vector NTI package (Invitrogen).

### Chromosome Preparation and Fluorescent *in situ* Hybridization

Chromosome samples were prepared essentially as described [11], [12]. To determine the integrated transgene *neo* in the genome, the p53GT22 clone was subjected to fluorescent *in situ* hybridization (FISH) as described [18]. The DNA fragment containing the STneo was obtained from pGTp53, labeled with biotinylated dUTP by using the Biotin-High Prime kit (Roche, Mannheim) and used as a probe for FISH on nuclei and metaphases. Chromosomes were counterstained with propidium iodide (Sigma, USA).

### Induced Cell Differentiation

MES1 cells and *p53*-targeted cell clones were subjected to EB formation in suspension culture in the presence of all-*trans* retinoic acid (RA; 10 µM, final) for induced differentiation [11], [12], [13]. Cell differentiation was monitored by phenotype and studied by RT-PCR analyses of expression of pluripotency and lineage-specific genes [12], [31].

### Cell Labeling

Cells were stably labeled by transfection with pCVpf or pCVpr followed by puromycin selection (1 µg/ml) and clonal expansion of GFP- or RFP-expressing colonies [16], [18]. Alternatively, cells were transiently labeled by staining with fluorescent dyes PKH26 (red; Sigma) or PKH67 (green; Sigma) for 3 min at final concentration of 2 µM in Diluent C, followed by rinses in PBS as described [35].

### Chimera Formation

Labeled MES1 cells and *p53*-targeted cell clones were transplanted alone or together into dechorionated blastula embryos of strain *af* and donor cells were monitored regularly as described [18], [21], [30]. Transplantation of medaka ES cells into zebrafish blastulae for interordinal chimeric formation was performed as described [35].

### Microscopy

Observation and photography on Leica MZFIII stereo microscope, Zeiss Axiovert invert and Axiovert upright microscopes were as described [12], [20], [21], [32], [44].

### Statistics

Statistical analyses were calculated by using Origin 6.1 software. Data consolidated were presented as mean ± SD and p values were calculated by using non-parametric student’s t-test.

## Supporting Information

Figure S1 **Detection of HR activity of medaka ES cell lines.** High HR activity of medaka ES cell lines. ES cell lines MES1 to 3 were transfected with pGTp53 (+) or pBluescript as mock transfection control (-), and subjected to PCR detection following 5 days of growth in the absence of drug or 14 days of growth under PNS.
(TIF)

Figure S2 **FISH detection of p53 gene targeting.** (A-C) Metaphases. (D-F) Nuclei. The FISH signal is green (arrows). Cells of clone #22 were hybridized with the fluorescein-labeled *neo* probe (green; probe 2 in Figure 1A) and stained for nuclei with propidium iodide (PI, red). The *neo* signal is highlighted by arrows. Scale bars, 10 µm.
(TIF)

Figure S3 **Junction sequence of targeted** ***p53*** **locus.** GT vector and targeted locus. *neo*, cassette expressing neomycin aminoglycoside phosphotransferase for resistance to G418. Paired arrows depict the position of PCR primers on the targeted *p53* locus. Sequences of five *p53*-targeted ES clones. The *p53* sequence included in the GT vector is shown in black. Shown here are only junction sequences. Shown in bold are ATG codon, polyA signal and stop codon introduced immediately downstream of ATG.
(TIF)

Figure S4 **Efficiency of Mitf-directed melanocyte differentiation.** MES1 and *p53*-targeted clones were transfected with pXmitf and pCVpf, the former expressing the *Xiphophorus* melanocyte-specific isoform of microphthalmia-associated transcription factor (mitf-M) and latter expressing pf, a fusion between the puromycin acetyltransferase and GFP. Following co-transfection, cells were pulse-selected with puromycin for 2 days to enrich for transgenic cells. At day 5 post transfection, cell counting was done on merged micrographs of living cells, and percent values of melanocytes and GFP-positive cells were derived by a comparison to the total number of ≥500 cells for MES1 and clones each.
(TIF)

Figure S5 **Distribution of MES1 and** ***p53*** **-targeted clone 22 in developing chimeras.** Following genetic labeling by plasmid transfection, parental MES1 (red) clone #22 (green) were mixed at 1∶1 ratio and transplanted at ∼200 cells per blastula. In total, 54 chimeras were daily scored from day 3 to 7 post fertilization.
(TIF)

File S1 **Table S1 & S2.**
(DOC)

File S2 (DOC)
