# Supplementary figures and images for: p53 Gene Targeting by Homologous Recombination in Fish ES Cells
Source: PLoS One. 2013 Mar 19;8(3):e59400. doi: 10.1371/journal.pone.0059400 (PMC3602087; doi:10.1371/journal.pone.0059400)

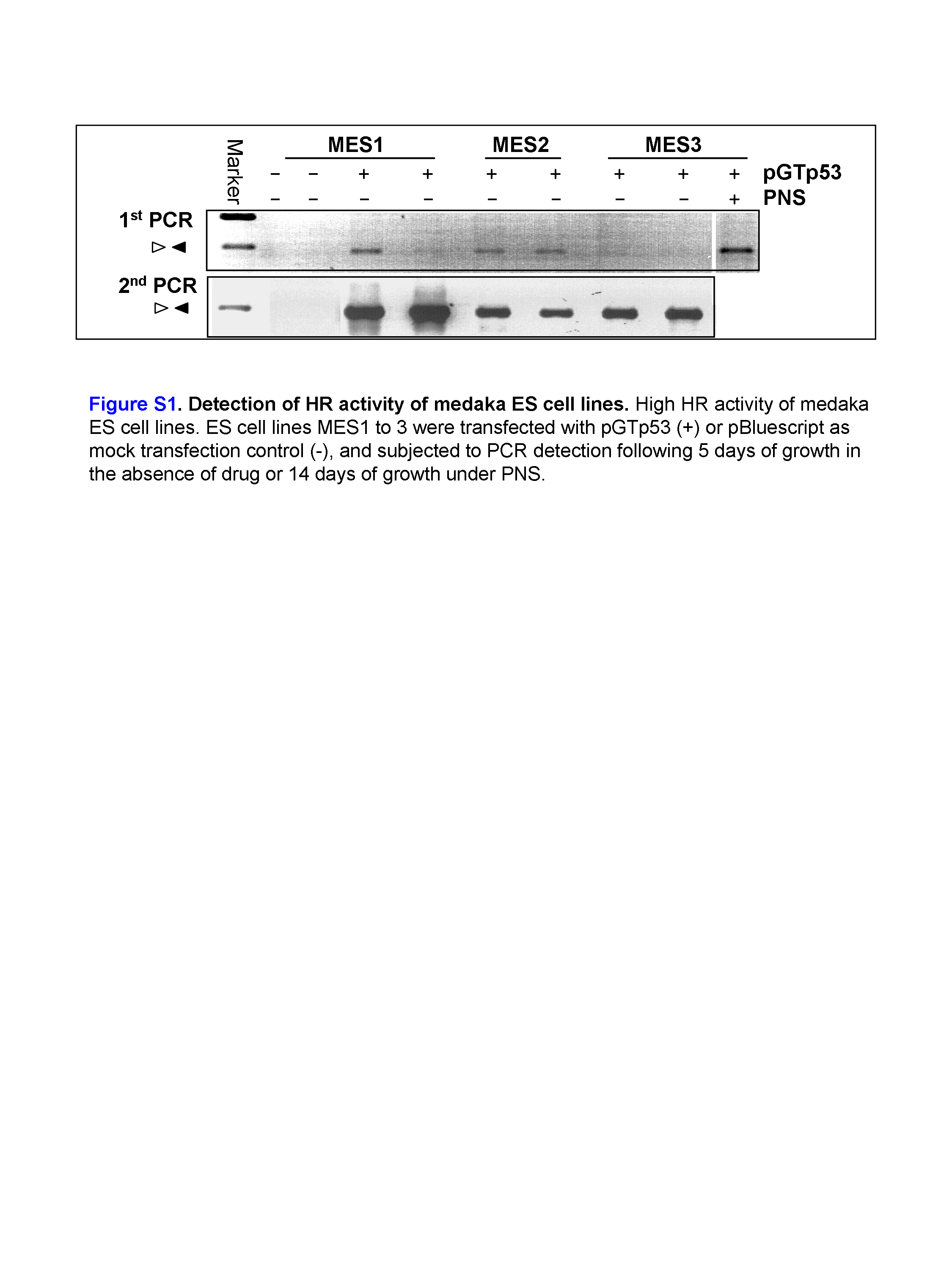

Supplement: Figure S1 — Detection of HR activity of medaka ES cell lines. High HR activity of medaka ES cell lines. ES cell lines MES1 to 3 were transfected with pGTp53 (+) or pBluescript as mock transfection control (-), and subjected to PCR detection following 5 days of growth in the absence of drug or 14 days of growth under PNS. (TIF) [file pone.0059400.s001.tif]

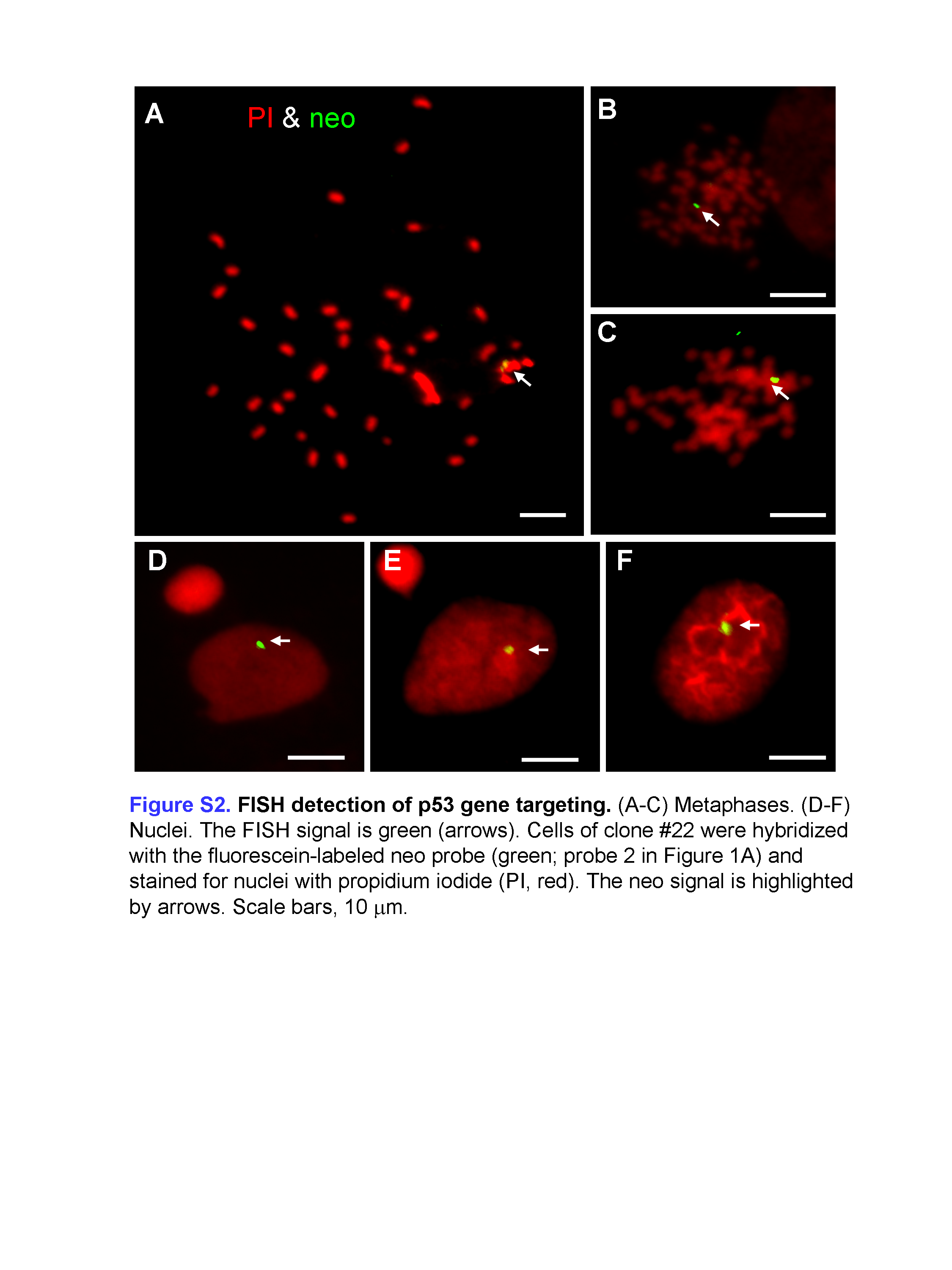

Supplement: Figure S2 — FISH detection of p53 gene targeting. (A-C) Metaphases. (D-F) Nuclei. The FISH signal is green (arrows). Cells of clone #22 were hybridized with the fluorescein-labeled neo probe (green; probe 2 in Figure 1A) and stained for nuclei with propidium iodide (PI, red). The neo signal is highlighted by arrows. Scale bars, 10 µm. (TIF) [file pone.0059400.s002.tif]

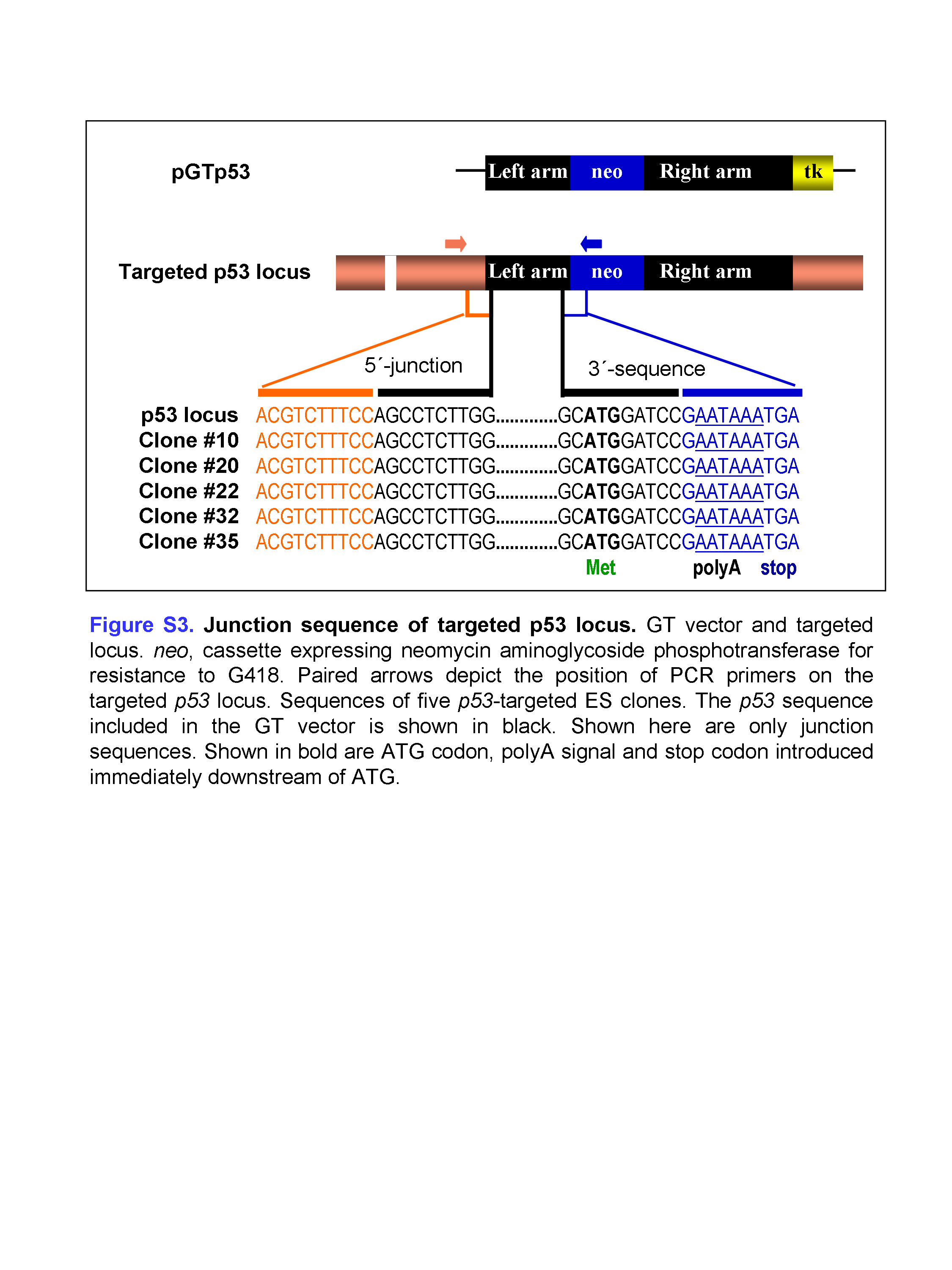

Supplement: Figure S3 — Junction sequence of targeted p53 locus. GT vector and targeted locus. neo, cassette expressing neomycin aminoglycoside phosphotransferase for resistance to G418. Paired arrows depict the position of PCR primers on the targeted p53 locus. Sequences of five p53-targeted ES clones. The p53 sequence included in the GT vector is shown in black. Shown here are only junction sequences. Shown in bold are ATG codon, polyA signal and stop codon introduced immediately downstream of ATG. (TIF) [file pone.0059400.s003.tif]

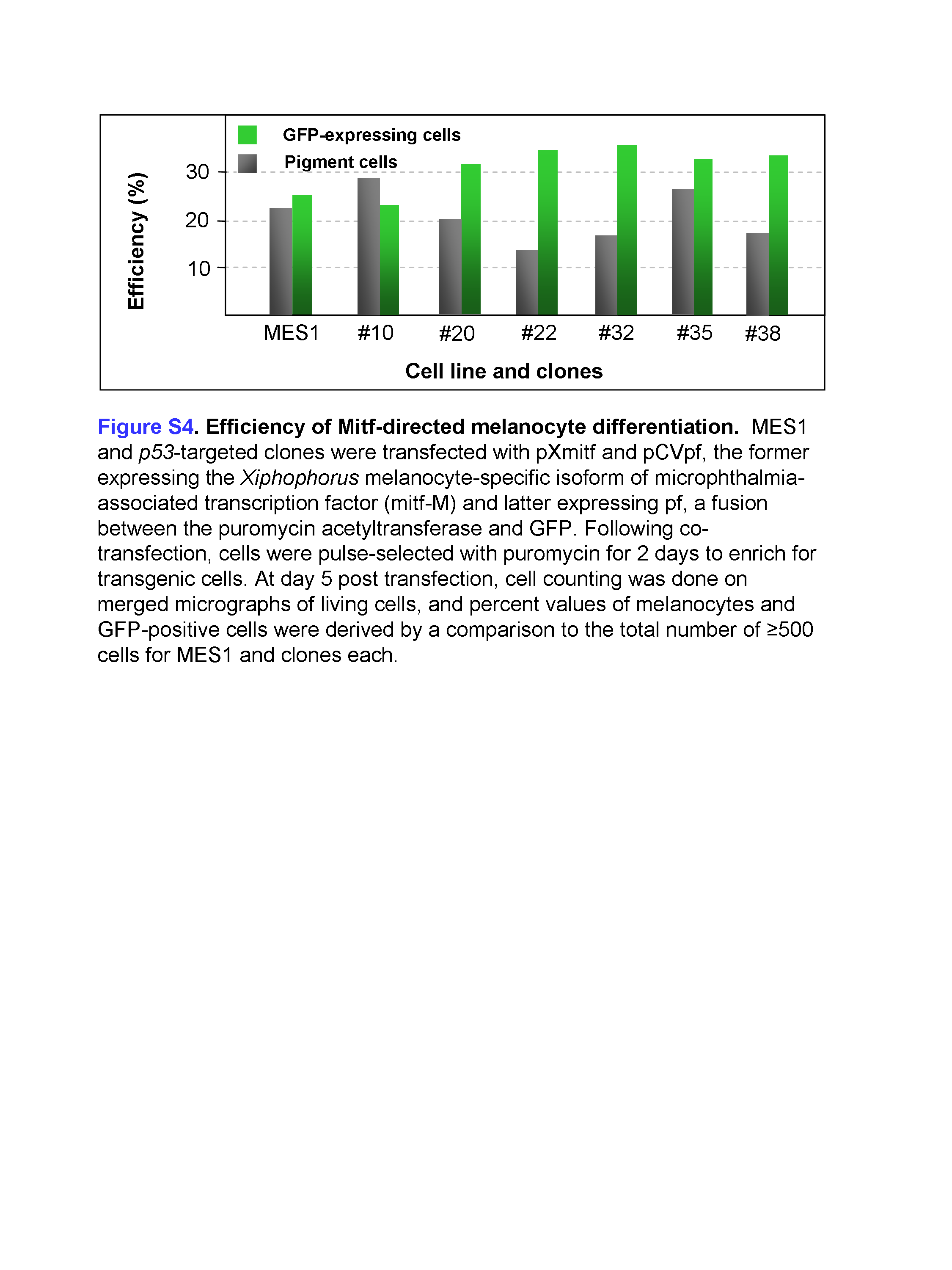

Supplement: Figure S4 — Efficiency of Mitf-directed melanocyte differentiation. MES1 and p53-targeted clones were transfected with pXmitf and pCVpf, the former expressing the Xiphophorus melanocyte-specific isoform of microphthalmia-associated transcription factor (mitf-M) and latter expressing pf, a fusion between the puromycin acetyltransferase and GFP. Following co-transfection, cells were pulse-selected with puromycin for 2 days to enrich for transgenic cells. At day 5 post transfection, cell counting was done on merged micrographs of living cells, and percent values of melanocytes and GFP-positive cells were derived by a comparison to the total number of ≥500 cells for MES1 and clones each. (TIF) [file pone.0059400.s004.tif]

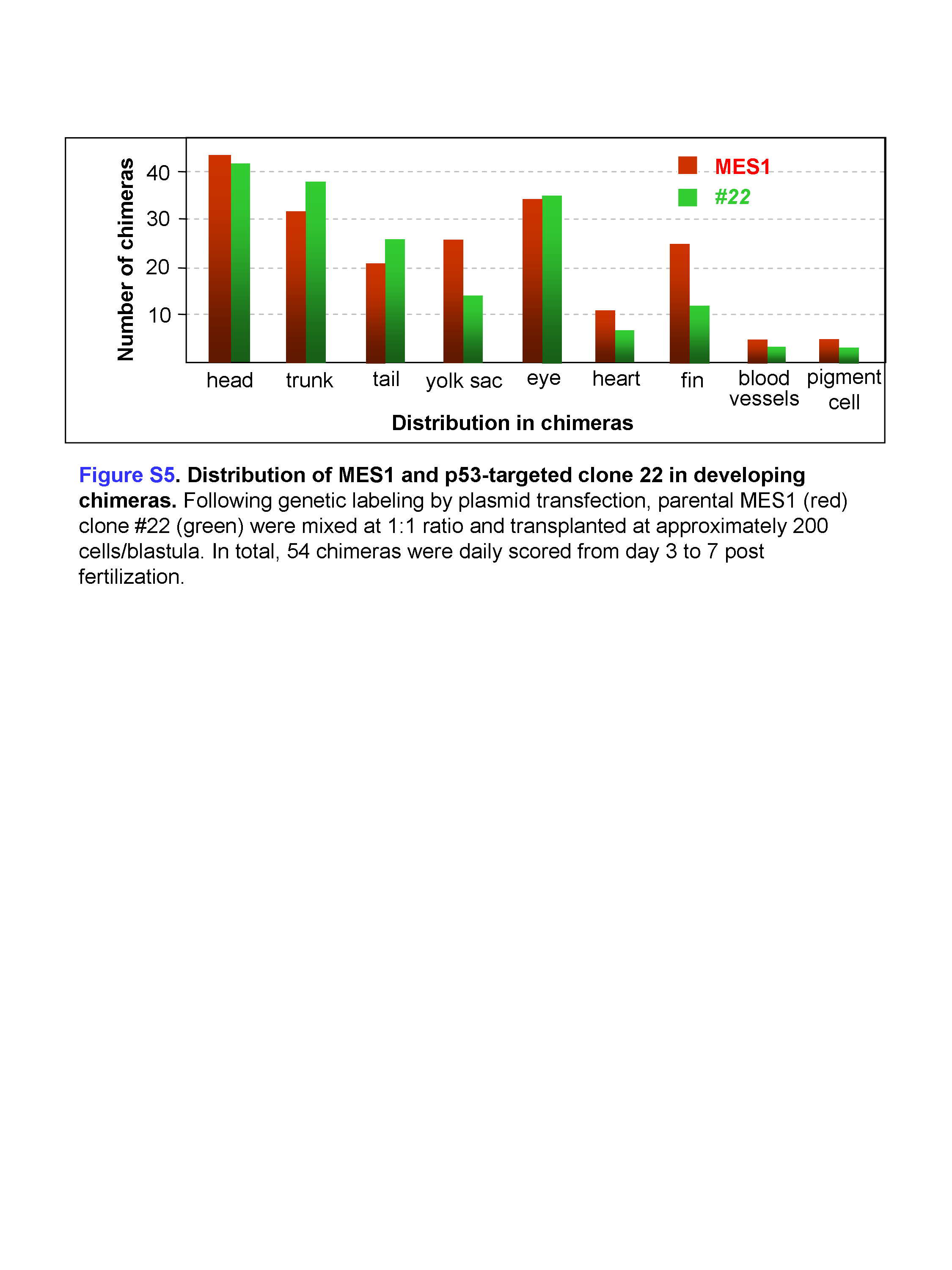

Supplement: Figure S5 — Distribution of MES1 and p53 -targeted clone 22 in developing chimeras. Following genetic labeling by plasmid transfection, parental MES1 (red) clone #22 (green) were mixed at 1∶1 ratio and transplanted at ∼200 cells per blastula. In total, 54 chimeras were daily scored from day 3 to 7 post fertilization. (TIF) [file pone.0059400.s005.tif]
