# Supplementary material for: p53 Gene Targeting by Homologous Recombination in Fish ES Cells
Source: PLoS One. 2013 Mar 19;8(3):e59400. doi: 10.1371/journal.pone.0059400 (PMC3602087; doi:10.1371/journal.pone.0059400)
Supplement: File S1 — Table S1 & S2. (DOC) [file pone.0059400.s006.doc]

**Table S1. Genes and primers used for genomic PCR and RT-PCR analyses**

| **Usage** | **Gene**  **(accession)** | **Primer name and sequence (position)** | **kb** | **notes** |
| --- | --- | --- | --- | --- |
| genotyping | targeted p53 allele | p53Ua: CGCCACCAATTGAGTAGCAGT (exon 1)  NeoRa: TGTCTGTTGTGCCCAGTCATAGC | 1.4 | 1^st^ PCR |
|  |  | p53Ub: TGATTGGACGACACAGGACGAAA ((38 bp downstream of p53Ua)  NeoRb: CGGAGAACCTGCGTGCAATCC (25 bp upstream of NeoRa) | 1.4 | 2^nd^ PCR |
| genotyping | wildtype p53 (ENSORLG0  0000006390) | p53F: TTATCCTCCGTTGGAAACTTTATCAC (exon 3)  p53R: GTACAGCTTATTTAAAGTTTCAGAGTA (exon 7) | 1.8 | 2^nd^ PCR |
| Southern | p53 gene | p53EXTF: GACATACTGTATTTCTGAAACCTC  p53EXTR: GAGATCGGTTGTGGTGCGTTC | 1.5 | External probe |
| RT-PCR | p53 cDNA (u57306.1) | p53ex6F: CTGTGGAGCACCGAAGCCATC  p53CDSR: ATTGCTCTTCAGCTTCTTCCCA | 0.55 | RT-PCR for inducibility |
|  | *Nanog* (FJ436046) | Ng1: ATGGCGGAGTGGAAAACTCAG  Ng2: ATGAACTCCAAGAATCCGGTG | 0.40 | Pluripotency marker |
|  | *oct4*  (AY639946) | Oc1: ATGTCTGACAGGCCGCACAGC  Oc2: AGTCGCGCCGGGTACCTGGCC | 0.32 |  |
|  | *klf4*  (ENSORLG  00000005643) | Klf4a: CAGAGGCGCTGATGATCTCTAG  Klf4b: TAAGTGCCTCTTCATGTGGAG | 0.34 |  |
|  | *c-myc*  (ENSORLG  00000007021) | Myc1a: GCAACCGCAAGTGTTCGAGTC  Myc1b: AGCTCGAGAGCCCTGCAGCTG | 0.30 |  |
|  | *Ronin* (ENSORLG  00000008903) | Ronin1: AACTGAGAAGCGACGAGTACTC  Ronin2: CATTTTCTTTCTGAAACCAAC | 0.30 |  |
|  | *sall4*  (ENSORLG  00000016130) | Sall4a1: CCAGCACTGCTGCCCCAGTCTC  Sall4a2: GGTATTTGTCTTTATGCCTCTG | 0.34 |  |
|  | *tcf3*  (HQ705658) | Tcf3a: ATGCCTCAACTGAACGGAGG  Tcf3b: CTGCAGAGCTGGGAACATCC | 0.43 |  |
|  | *zfp281a*  (ENSORLG  00000002799) | Zfp281a1: TTACACACTGGTCTCCTCCCAG  Zfp281a2: TCTGTAGCTTTGGCTTGTTGG | 0.26 |  |
|  | *nf200* (ENSG  00000100285) | Nf200a: AACCTGCCGCAGCCAAAGAACC  Nf200b: GATTTAGGCACAGGCTTCTCTG | 0.28 | Germ layer & differenti-ation marker |
|  | *eed*  (ENSORLG00000000063) | Ee1:ATGAGGGAAAATAAGAGCATG  Ee2:CGGGAAGTGGATCTTCTGGGA | 0.8 |  |
|  | *gfap*  (ENSORLG00000002346) | Gp1:ATGGGGAGTCCAGGGGATCGG  Gp2:TCCCTCTCGATCTCCAGTCGA | 0.3 |  |
|  | *actinin2* (ENSORLG  00000005187) | Actn21: GTATAATGATTCTGGTGGATCC  Actn22: CATAGAGGGCAGTAGAGAATGC | 0.28 |  |
|  | *ntl* (ENSORLG  00000011262) | T1: ATGAGCGCGTCGAACCCGGAC  T2: AGACGGGCGCTTTCATCCAGT | 0.40 |  |
|  | *Myf5*  (ENSORLG0000001625) | Mf1:TTCTTCTCCCAGCCATGGATG  Mf1:TTCTTCTCCCAGCCATGGATG | 0.23 |  |
|  | *sox17* (ENSORLG  00000011542) | 17a: ATGAGTAGTCCCGATGCGGG  17b: GCCGGAGTCCAGCCTCTTAAT | 0.45 |  |
|  | *mitf1* XM_004068600 | Mi1:CCATGTTGGAGATGTTGGAAT  Mi2:GGACAGCGTTAGTCCTTGATT | 0.4 |  |
|  | *β-actin*  (D89627) | Am1: TTCAACAGCCCTGCCATGTA  Am2: CCTCCAATCCAGACAGAGTATT | 0.65 | Loading control |

**Table S2. Gene targeting efficiency at the p53 locus in medaka ES cells** ^1)^

| Batch | Colony  n | Colonies PCR-assayed ^2)^ | | Colonies Southern-assayed ^3)^ | | % GT efficacy ^4)^ |
| --- | --- | --- | --- | --- | --- | --- |
|  |  | total | positive, n (%) | total | positive, n (%) |  |
| 1. | 24 | 23 | 9 (39.1) | 6 | 3 (50.0) | 19.6 |
| 2. | 16 | 16 | 10 (62.5) | 5 | 3 (60.0) | 37.5 |
| Total | 40 | 39 | 19 (48.7) | 11 | 6 (54.5) | 26.6 |

^1)^ Shown here were the last two experiments.

^2)^ Positive refers to colonies that were positive for genomic PCR by using a primer pair specific to the targeted locus.

^3)^ Only PCR-positive colonies were examined. Positive here refers to the appearance of a 9253-bp band for the targeted locus.

^4)^ Derived by multiplication of % PCR assay and % Southern assay.
