# Supplementary material for: p53 Gene Targeting by Homologous Recombination in Fish ES Cells
Source: PLoS One. 2013 Mar 19;8(3):e59400. doi: 10.1371/journal.pone.0059400 (PMC3602087; doi:10.1371/journal.pone.0059400)
Supplement: File S2 — (DOC) [file pone.0059400.s007.doc]

**Ol p53 (ensembl location: medaka 18:14435616:14502592:1) 66,376 bp**

[**http://www.ensembl.org/Oryzias_latipes/Location/View?db=core;g=ENSORLG00000006390;r=18:14435616-14501992**](http://www.ensembl.org/Oryzias_latipes/Location/View?db=core;g=ENSORLG00000006390;r=18:14435616-14501992)

**To analyze the EcoRI sites (for designing a proper 5’- external probe for Southern blot), a 30-kb upstream sequence of the of Ol p53 gene is shown as below:**

1 GGAAAAGGATACATTTTTCTTATATATGTCCTCCAGTATCAGAAATATGCCACAAGAACATGATCAAAACACCATAAATATGTTTTTCACTTGAGATGGT

101 CTTAGTTGATTTGCTTTGGGTTTTTTTCCTCTGACAGAAAAATCCTTTGCTATTTATTTTATTTTAGTGTCATAATATATACAAAGTGAAACAAAATTTC

201 TTTCTATTTTCATATTTTTTTTACTTTTTATTTTAATGCTATCCATTCTATTTCCATTTCTTCATAAATGTATTAATTGGCTGCATGTATGAAAGTCACC

301 AAACAGATTTAGGCTGCAGCTGGTAAGGGGAATCTTGGGTAAAGAAATCTTGAATCTTAAAAAAAAGTGATAGGCCGTCTAATACCCTTCAGTCCTACTG

401 ACCACGTTTGGCAAGATGCTCCTGAGAGCGGATGATGTAAAGGAATCGAGGGCTTTCGGGGCAGAAAGGCAGCAGACCCATCTGAAGTACAGTTGGCATC

501 ACTGTTAAACCCAGCAGAACCGGCCAAAGGTCCTCACTGCCGAGTAAAGACTCCAGACCCAGGATCTGTTTCGAAGATAAGTATTTAAAACACGCAGCGT

601 TTGAGAAGTTCTGAAAAAGATTTATCTCTGCAGCAAAGGATGCAAAGAGATTTTTATTTGATCAAATACAGCCATTTATCTTACATTAAGAGATAATAAT

701 AAAGTTGTTTTTAGGAAACAGAGTAGCCACATTGTACCTGTGCTATCAGAATGCCTGTGACTATTCCCAGTTGATGCAGGGTACCCAGAGCCCCCCGAAG

801 ACTTGTTGGTGCAATTTCTCCAACATACATTGGTGTCAGGGAACACGCCAGCCCTAAAAAGAGAAACAGATGAAACCATTTGCAAAAAAAAAAAAAAATC

901 ATATTCGGTAAAATAGGGANNNNNNNNNNGGTGGAACCCTTTTCAAAAAAAAAAAAAGATCATATTCTGTAAAATATGGAAAATTTTAAAAACAAATTGC

1001 ACCATTTTACTGTATCTCTTACCACAATAGGCACCAATAATGAAACGTCCAAGAATCATCATTTCAAAGGAACGACAGAGCTTGGACATTCCCATCATGC

1101 TCCCACCAATGAAGGCAAACAAGTTGTTTATAAGCATTGCTTTTCTCCTGGAAGAAAGAAATAGGCACATCTTTTCTATATGTTAATTTAAAAGTATACA

1201 TTCAGTCATTTGAAACTGCTGACAAGCAGTTTGAGCCTCCTCATCGTGTGAGAGGTTTGATCCTTTAGAGGGCGCTGTCTTACTGACTGTTGTAGTGTTG

1301 TGATTGGATGTTTAAGGGGTCTGGAAGGTTTTTGTCATGGGTCTACACTGCAAGAACACAAAATCTCACCAAGTATTTTTGTCTGGTTTCTAGCCTAAAC

1401 ATCTTATTAAACTGTCCATAAACATGCTCAATTGGCAACTCTAAATTGTCCGTGAGTGTGAGAGTGAATGGGTGATTGATATGTGGCCCTGGCCCTGTGA

1501 CAGACTGGCGACCTGTCCAGGGTGTCCCCTGCCTTCGCCCGTAAGTGGCCGGGATTGGCAGCCCCAAGACTCCGTAAAAAGGTATAAGATGAATGAATGA

1601 CTGTAAGAGTCAAAAGGTGCATTCAAACTATTAGTTTTTTTTTACAACTGCAGTTTACAGTTCTACTTGAGCCTCACCAAAACTTGTAATTCCTCACTGA

1701 AAAAGAGCAACTTCAGCTTGTTTTGCTGTGTTCAAGCTTTTGATTATATTTTTTAAATAAAATAAAATCAAGCTTTATTTTATACTGCACTTTTCCTATA

1801 AAAACCCCACACAAATAGCTTTAAATACGTATAATTTGTCATCTTTTGTTGATTTAATAGTCAATTTGTAAAAAACAAAAAGGTTTTTTTTTTCTTTTTA

1901 ATCATAATTCCTTTAGTGTGTAATATTTGGCCACCCTTTCGTGGCTTTCTGTTCAAACCTGCTTCCCACCACCTGAGAAAAAATCTTGAAAAAAAGCCTC

2001 TGGACTTCTCTCATTTGAACAGATTGGAGTACATTTTTCGCACATAGACCCTTTTATTTAACTCAAGTCTAACATGTGGTACATTAATTTGGCAAAGAAT

2101 GCTTGAAAAACATGACTTTTATGATGCAATTTCTATACGGTACCAAACTTCCTTAACGCTTCTCACAGTCAAACCAGAAAGCTTCCCTAAAAGCTTCACC

2201 TCCTCCTTAACCCACTGGAGAGATGTCGTTGGCGACACCTAAATAACACACCCTCTTTGACATACTGTAAATCTTTGACCGTTTACGCACCGGGATTCTG

2301 CTGATTTTGACACACAGTAACTGTTTTTTAAGTCGACTTATCCCAATATGCATCACGGCTCAAAGCCACTTTTCTCCAGGACAGAATCATCTGATTTGCA

2401 CTGATTGCGTTAACACTTAACTATTGTAAAGTGGTGTCATAATCTTTTTACTTTTCCCTTGTTACTGCTAATTCCGCCTGCATCCGCACTGTGGTAAACT

2501 GCACCAGAGTTCACTTACAAGTGAACCAACAAGTGAAGCCTCATTTTAAGGTGGGCCAGGTTCAGATGAGGACTGCACCAATCTGGCCTTAAACATTTAC

2601 ATTGTTTGGTTTTGGAAAAAAGTTCAAGGATTGAATACTGGAATCTATTGGTTCATTTTATCTAATGTAATATTTCTTAAACAATGGATTGCAGCTAATC

2701 TGCATTATGAAAAAGTTCTTCTTTGGCCAAAATTTGTAAAACAAAAAGGGTACTGTTATCTAAGTTATACATAAATTAATTAAGCAATTTTTAGAAGATC

2801 TGTAGGCTAAATATGCAAGAAAAATATATCTATATCAAAATTTAAAAAGTGGAATCAAACCATCCCTAACTTGTTTTTACCACACAAGTGGTATTGTTTA

2901 ACTAAAGCAGAAATAAACTGTACATTTTAAACAACTAAGCTAAATGGAAAAAAATTTGAATAAAAATAATTATAACAGATGATGGAATTGATGAGAGGCC

3001 CTTGAGAGCAATTATAACTCTATCTATCTATCTATCTATCTATCTATCTATCTATCTATCTATCTATCTATCTATCTATCTATCTATCTATCTATCTATC

3101 TATCTATCTATCTATCTATCTATCTATCTATCTATCTATCTATCTATCTATCTATCTATCTATCTATCTATCTATCTATCTATCTATCTATCTATCTATC

3201 TATCTATCTATCTATCTGACAAACCATCCATCCATCCATTGACTGTATATGAGAACTGTACTGAGTAACTCCTCCTCCCTGGTGTCCAAAACAGGAAGTA

3301 GCCGCTGATTCCAAGAAGCCAAAATCCCAAAGACTTCTAAAGAGAAATACTAAGAAGTCATTCTTTTTGTCACAACAACCATTGTTGTTGTGACAACAAT

3401 GGTTGTGTTGTTGTTACGATAAAATTTTAAACCTCTCTCTATGTTTTATCCCCCAAACAATGAGAGCTGCTGTCAATGACCATATCATGTGCTAAGGCTC

3501 AGGACATTTTGACCTCAAAGGTCACTGCAGTTTCAACCCCAGCGACAGCTGCTTTCAATTAGAAAGTTCTGTCTCTGACTGTGTCCATGATGAACATTTC

3601 CACTCGCACCTCTCCACTGCCAACTCTGCTAAGATCTCCTTTCAGTCTCGTTGGAGGCGGTGGGCCACAGTCCTGTTTGAGTCCCAGCTCAGGTTACAGC

3701 GGGAAACTGGGTCGCTTCCCTCTGTGTGACCAGGATGGGAGTGAGGAATGGCTTTGGATTGTAATGGAGGAAAGCATTTTCCATCAGGGACTTTCTCTAT

3801 TTTTCTATTTCCCCCCCACCTGACTAGTTAGCTTTATTTCATCCAAATCTCTTCCTGGTCTTTACCCACTTATAGGGAAATCAAATTTAGTTTTACTCAC

3901 AAGTAATAGAAGTGGAGCCTAAAGATACTTGAATTTACATTTTCCCACTTTACTTCAAGGTTAATTGGTACATTCCAGGAAAAACTTTTTCCTACCTGCC

4001 CAACCATTCAGAGATGAAACCAACGCAGAAGGAGGAGAACATGCCTCCAATGGAGAAGATGGCAACAGACAGAGACCAGAGGGAGGTGAGGGTCGCTGTG

4101 GGGATGGGCTCTCCATACCGATGCACCCATGTCGCGTTGTAGTCCTCCTCGATGATCTACAGAGCAACATTACTGGTCAGAGAGAATTTATTCAAAGGTT

4201 GTTTTAGTCTAACACCTGAATTTGATCATTAAATTATTTTCCAGTCACAAAGTTATGGGTCATACCACAACTTTTTCAAAATTGTTTTTGCAATTAGAGC

4301 TTCAAGCTTAAAGTTAGATAAATAATAATTTTGAGCAACAGAGAGTTAAGAAGCACTATACTGATATAAAAAATGCACCTTAAATATGATTTAAAGGTTG

4401 TACAATGAGTTCAGAAGGGTTAACGCTTGAAGTTTAAACTAAAACAATTTTTTTTAAATGTACTGGTTATTTAATTGTACTGGTCTTTTTTCATGGTTAG

4501 TGAATACCATGCATCCCAAACTAGGCTAAAAACTACATATTCCAACAGAAAGGGCTAAGAAAGCAAATGATAAAAGTCTCAGCAGCCCAGTTTTTATTTT

4601 GGTGTTCGTCTGTTTGTAGTAAACTAGGTTTCTGAGCAGATCCCTGAATACCAGCAGCCACGAGGAGCCAGTCTGCCGCTCCTGTGTGCACGCGCACATA

4701 TTGTCATGAGAAGCAAAACCAAATTGCCATTTTTGCTAGATTACCTGAGTATCAATGTTTGACAGTTTTCAGATGCCCCCCCCCCCCCCCCCCTTTTTTT

4801 AAGGGCCNNNNNNNNNNNNNNNNNNNNNNNNNNNNNNNNNNNNNNNNNNNNNNNNNNNNNNNNNNNNNNNNNNNNNNNNNNNNNNNNNNNNNNNNNNNNN

4901 NNNNNNNNNNNNNNNNNNNNNNNNNNNNNNNNNNNNNNNNNNNNNNNNNNNNNNNNNNNNNNNNNNNNNNNNNNNNNNNNNNNNNNNNNNNNNNNNNNNN

5001 NNNNNNNNNNNNNNNNNNNNNNNNNNNNNNNNNNNNNNNNNNNNNNNNNNNNNNNNNNNNNNNNNNNNNNNNNNNNNNNNNNNNNNNNNNNNNNNNNNNN

5101 NNNNNNNNNNNNNNNNNNNNNNNNNNNNNNNNNNNNNNNNNNNNNNNNNNNNNNNNNNNNNNNNNNNNNNNNNNNNNNNNNNNNNNNNNNNNNNNNNNNN

5201 NNNNNNNNNNNNNNNNNNNNNNNNNNNNNNNNNNNNNNNNNNNNNNNNNNNNNNNNNNNNNNNNNNNNNNNNNNNNNNNNNNNNNNNNNNNNNNNNNNNN

5301 NNNNNNNNNNNNNNNNNNNNNNNNNNNNNNNNNNNNNNNNNNNNNNNNNNNNNNNNNNNNNNNNNNNNNNNNNNNNNNNNNNNNNNNNNNNNNNNNNNNN

5401 NNNNNNNNNNNNNNNNNNNNNNNNNNNNNNNNNNNNNNNNNNNNNNNNNNNNNNNNNNNNNNNNNNNNNNNNNNNNNNNNNNNNNNNNNNNNNNNNNNNN

5501 NNNNNNNNNNNNNNNNNNNNNNNNNNNNNNNNNNNNNNNNNNNNNNNNNNNNNNNNNNNNNNNNNNNNNNNNNNNNNNNNNNNNNNNNNNNNNNNNNNNN

5601 NNNNNNNNNNNNNNNNNNNNNNNNNNNNNNNNNNNNNNNNNNNNNNNNNNNNNNNNNNNNNNNNNNNNNNNNNNNNNNNNNNNNNNNNNNNNNNNNNNNN

5701 NNNNNNNNNNNNNNNNNNNNNNNNNNNNNNNNNNNNNNNNNNNNNNNNNNNNNNNNNNNNNNNNNNNNNNNNNNNNNNNNNNNNNNNNNNNNNNNNNNNN

5801 NNNNNNNNNNNNNNNNNNNNNNNNNNNNNNNNNNNNNNNNNNNNNNNNNNNNNNNNNNNNNNNNNNNNNNNNNNNNNNNNNNNNNNNNNNNNNNNNNNNN

5901 NNNNNNNNNNNNNNNNNNNNNNNNTGTCCTTTTTCCAAAATTGAAGGCATCATGGTGGTATATAAACAATTAGCAAAACAACCAAAAATATTATTCCCAT

6001 TCCATTACAACAGCTTGAAAATTATACTACAAATGACCCTTGTTGACGTAGGAGGCGAACATACAACATAATAGGGCGCCATCCGATAAGTTAACAACGT

6101 GAAGGGGTCCATCTGATTGGTCAAATGCAGAAAGGGATTCTTTCAATCCGTTAAACACTTCAAGCTGCTATAAGATTGTTTGGCCACATCTAACCATTTA

6201 TTGCAAATTTGCTGTCTTTTGCGATATGCATATTGCACAGCTTGAAATTGCGATAACGATAAATTTGCGGTATATTGTGCAGGCCTAGTTAAAAGCATCT

6301 CATTTAATCAACATCTTAAATTTGATCCACACGTTTAGCTTGGTGTGATTAAAGAATTAAACTTGGCATTCTTTCAATACAGCAAGTAGTCATTTGAAGA

6401 AATAAAAAACAATAGATGAGTTATTCTCACTATAAAGTTTTAAAAAATCAGCAAAACAGGAACAGCCAAAGTTTTTTTTTGAGATTTCACGGAAGCAGCC

6501 ATTTGGAGATGAGCAGAAAAAGCCCTTCTACTGCCCCTAGTGGAACGATAATGAACTACAGGGCAGAAAACATGGACTAAGGCTGTGTCCGAATTCCCAC

6601 CCTAACCCCTAACAACTAAAAAACTATATAGTGCGGGACTATGTTGTGCCCTGGATTTTAAAAGCAATTCGGACACCATGCTCACTACTTTTTTTTTCTT

6701 TTTTAAACGCGAATATGACATCAATTTCACAAAGGTAAAATTGAATTGATATGAGCGTTTTGCAATGATCATTTATGAATCCGCTGTCTTCTGAAGACAA

6801 AAATATTGATGGCTTATTATTTGCAAAAACTGTCCAGACGCAATGCATTGTGGTCTGTATTCAACGATCTAATGAACATCGATGCACATTGGTTTTTCGC

6901 AGAGACTCCTGGGAAATGTTTTAGGACACTCGATTTTGGAATTGCAGATTCAGACAGCGCTACAAAATGGCGAACCCACTATATAGTGCAATATATAATG

7001 AGTGGGGAAGGAATTCAGACACAACCCAAGTTATATACAATGGGTTCTTATGTAAAAGTTTTGATTATGCAAATGAGATAGGGGTCTCAGAACTGCATCT

7101 ATCAAAGATGATGGGAATGGTTATATTGGGTTAATTTTCTTGCCACATATTTAGTTGTTTCAGTATATTTATTTTTTTCTACCTTCTGAGGTGCATTGAT

7201 GACTCCAATGTTGTACCCAAACGTTAGAGATCCCAGAACAGCAGTGAAGACTGAGAGTGCAAGGGTTCCAGTCACCGTCTGGGGAAGCACAACAAAGACA

7301 GGATTTGAACAAAGTCAAGACGCCCTCATTTTCAGAGCCAAAGCTGAAACTCTCTAATTGGAATTAAGGCTCATTCAAATGAGTTTCCCTGCTCTACAAG

7401 CGTCAACTATATATAACTATGATCCCGACGAGAGACAATCCCTTCATGACTTCACTGGCTACTCGCCTGAACTTTAGCTTCCCCTCTAGTTTCCCATCAG

7501 GCCTCAGACACCTGCCCTCACAGTGACAGATGAGATGACCTTGGGGAAACTGGTTCCTCCCGTCGAGCAATGGCTTTAATTTTCCATAAGGAGCAGCATT

7601 TTATAAGACTAATAAGTTACTGTTGTACAAGTTAGCAAAAAAATAGAAAACAGAGGCTTGACTTGATGTGATTGTTCCCTGATGAGATCTGCTGGCTAAC

7701 AGAGAAGGTGTCATAGCAACATACAGTTAACATGTCATGGACCTGATCAAAACACTGGAATTTTTCTGGAATTACGTTGAAGATGTTTTGATCCAATTAT

7801 GGGAATGGTTGTGATCTCTGCTTGTTGAGTTTTCAAGCTTTTTACAGCTGAGCCACTTAAATTTTGTAAATGATTGAACAGATTCTGACCAACCCCCAGC

7901 CTTGAGTGTCTTGCCCTCAATGTTTTGACTCCATGGTAGTTTAGTCTTCAAGACCCGAAACAGACATGTGTCTGTTGTGGTTTGGGTTAAGTGTCAGAAG

8001 CAGCCGGTTGAGCTGTCACAACAGCATTTCACCTCAACGACTGCGTGGTCATTCATTTATGAACAGGAAGTCACCTTTATCTGCCTCGGGCTATGTGTCG

8101 CGGTCAGGTAACCAAATGTCATAAAAATTTCCTTTTTTTTGTTCTCTGCATCTTTGGTTTATTGGAGCAGAAAGCAGTTAATGAGACATTTAGCAGGACG

8201 CCAATACAGATAGAATTGAAGATTAGATATTTCATCATCAGAGACGAGGAGGAGTCTGATTATTTAAAGTGACCCTTATTGATCACTATTTAGGCGATTT

8301 CTTCACAACAGCCAAAGGTGGTAAAACTTGGCCAAACAGGCTGTGCTGCAACACTGGAGTTTGATATGTACACTGAGAAATTTGACAGGTCAAGGTGAGA

8401 TTCTTCTCCTTATTTAGGATGGATCATTCCTTTTTTGGTATTTTTCCTTAAAAAATTAATTTAAAGCTACTGCAGAAAACCAGAGCTGTGAATCTTATAG

8501 TAAGACATTATTTCTTTTCATTTGGAGACTCCTATGCAAAAAAAGCCATGAATCAGCACGCCAAAAACGCATGACCTTTCAGAGGCACCTTTGTGAGTCA

8601 GGTCTTCCTGCCCAAATGGGCCCATCCATTTCGCAACAAGGCAAAGACTTAAAGCATACCTCCCCTCCAAGCTGCTGAAACCCTGCCGGCATGACTCTGG

8701 CAGCTCCTGGCACCCAGTGGCAGTTGGTGACAAATGTCTCTGCTGCCAGATGGAGTAAAAAAACTCTTGTAACCTTTGAGCACTTTGCCACCAAAAACCC

8801 GGGCAGGCTGATTAAATCCAAACGAGAAGCCCAAAGACAGAAGCTCTGGTGTGTGCTGTACTTCTGCTTGCCTTTGAAGTTTGAAGTTTGTGTTGTGCGT

8901 AAAGCTGAGCTCGAGTGACACCCACGTGCACGCCTTCGGTGCACGCAGGAGAGTTGAGGACAGACGGAGGGAGGGGGTTAAGATGGTGGGTGGTGGGACC

9001 AAGGAGGAAGCTGTCTTGACCTTAGCTAGGATTGGCTGATGGGGAGAGCAAATGTTCAGAGACAAGAAAGTGGAAGAACACAGAAAGGAGAACTTTGGAG

9101 ATAGATGACAAGGAGGGTGATAAGACTGTAACATGGATTTTGCAATGACAGAAGTTACTTTTCTGAGCTCAATGTCAAAATGCAAGCTTAATGTTTCATA

9201 AATTGTATGAAGGTAGGCTGTGGCTGAATGCATAACTCTTTAAAATGCAAGATCTCAATCTTGCATCTCATTGAGATCACGACATTGAAACTGGATGGAA

9301 ACTGGCTGCAGGTTTGTCATTATATTCAGTTAATTGACTTTTGGAGAATCGGAATCCGATTTCATGAGTCAACATTTCTGTGCAGCAAAGAGGTGGGCTC

9401 GTCAGCCGTGCTGGCGGCCATTGTCTGTCCTCCCAAAGGTCTGAAATCCCTTTCTATTTCAGACCGCATGCCAAAGAGGTCGTACACCTCTGCCCAGCCT

9501 GTCTGCAAACCTTTGCAGCTCCTCCTCTTCTGTCGCGTTCAACAACGCTGAAAGACCCACTCCAATGAAAATCAGGCTTTTGCTGTTTTTAACATGTTCG

9601 TTAAGCATTTTTCTGATGGACACGTATCAAGAAAATTAAGCCTAAAATTGCGTTTATGAGGATTTCTTTATTCAAATCGTTGAGAATCAGGAGCAGATGA

9701 AAAAATGCAGTTTGTAAAAGCTGCCGCTCTGCAGAAACTATGTCCTAGAAAACGACACAGGTTTTTTAACATTTTGGCTGAAAACAGCATAATTAAAAGA

9801 CCACTTAGAACGCTTTTACAATAGATCAAAAGATGATCGGAGTGGGACTTTAACCCCCCTTAACAGGTGATCTCTAAATTGAGTGGCGGGGGTGTGGTCC

9901 GGGGGGGGAGTTAGAGACCAAACTGTCTGTGGATCTGATGGGTTGGGGTGGGGGTGTTTAATGAGATCAGCCAAAAAGAGTCCTGTGGGACAATGTCTCC

10001 TCAACACCCTTTGGTAACATAACTCTGGCCTGCCCCAAAGCGGACACACCCCCTCACCTCACGCCACTCTTTTCTTGGAGCTCATTGCCTGTCCATCTTT

10101 TTTTGTGGGAAAACATGACACCCCCTCAACTGAAGCCAGGGCCCCCCAGCAGAGTCATGTGTGACTCCAACTAAATGCAGAGAAACTTTACACCCCCAGA

10201 ACAGTGGTGTGGAGCCCCTGCCAACATAGTTTGAGAGTTTATGCGCACAACCAAGAAAAGAAAGATGAGCTCCAAGAGGATATCGTGAAATTCAAGGGGG

10301 GGACAGGGAAAGGAGGGATGAATGCAGACTTAAATAGCATTTTGAATGTTCCTGGCAGGCGACGTTTCAGAGGACAAAATGTCAGTCGTGAAGTTTCCGA

10401 GCCAAAGGCGTCACACACACCCACACACATCATGTGATCGCTCTGTGGTGATGCAGAGAAACAGCCTGTGTAAATAATATTCCACCTTGTCGCGACCTAT

10501 ATTTAACAAGTGTTTTGGTGTGTGTTTAGGTGTGTTCTTTGGGGTCATCTGGTTCGAAATCATGCTACAGCACAGTCGCGTGATGAAGGGCAGGTGTTGA

10601 AAGTGTACCGATCAGACATTTACACCAACATGTACTGTTTGTCCCAATGTATTATGGGTAAAAAACAACAACAGAGCCAGTAAGTTTCAAAATATACCAC

10701 TGTATCCTCATGCCAAATTAGGGTTAAATCATAGTATTTATATATGGACAATTATTTTTCAATATACTACCCTTTTTATTATTATATTTATCATAATTAA

10801 GGTTGTGTTTTGATTATTTTTCACACTTTTTTAAATATATAATTATTATGATGTTTGTTATTACTCCTGGAAGTTGTTGTGGGGCACTACTGGCTTCGCC

10901 ATTGGGGGGTACTGTAGCTGCAGTCCTCGTCCTCATTAGGCCGGCTGGAGTCCCGCTCTGTGCTCTGATAGCCGTAGAGTGGGCCCTGAGCTGCTGTGTT

11001 GGCCGGGCGCTGTGGCAATGGGCCCCGTGGTCGCCTTGGAATAAGTGGATCCCCACTGTATCGTTTCTGCACAGCAGTACAGAGCCAGACTGTTTCTTTA

11101 CGCTGCAGTTTTTATGTTTTTGTTCAGTTTTGATGTAAGGGTCATATAGGGGTAACTGGGTGGGTGGAGGGAAGAATCTGGTGAGGGAGGGGGGCCAACT

11201 GTGTGTGATTGTCCTGTGTTTTCTGTTTTAAATAATTTTCATCTTTTATGACTATTTTGTTTGCTTTTATGTAAAGCGCCTTGGGTTTTCAACCTGAATG

11301 AAAGGCGCCACACAAACAAATAAAGTTAAGTTTGAAGGTTTGTAAAAAATATAGGTTTAGACTCTAACTTGACAGAAAATGTATGTCACATCAGTCCTTT

11401 GATTTTATTTAGTTAATTTATTTTAATTTGATTTGATTACTAAAAAATAATTGAATACTGAAATAAACAAAAAATTAGAAAACTCAAATACTTCAACAAA

11501 TTTCACATTTCAGTTATTACATTGATTACATTGAATACAAAGAAAAATTGATGAAAATTGAAAGAAAATATTTAATCTTGTTGACGGTGGATATACCCAT

11601 AATAAGAAATAGACTTTTTGCAAAAAGGAAAACAGCACAGAATTTGAATATTTGGAATCTATTTTCTATTGCTGGATTTCAAATTTCTCAATAAGTCTTG

11701 AGAAATCCTTTTTCTTAGAATACTTAAAAAAAGAATAGAATGCTGAGTTCTATTAGATTCAAGTTTTAGCTTTTACAGACCCAGATTAAAAATAGAAATG

11801 TTTGGCCTTTTTAAAATTGAAACTCAAAACTTTTGTGCTGTTAGCAAAAAGGCATTAAAACAAACTCTTGAATAAAAAACCTCTTCAACTTAATAAAAAC

11901 AGAGTCGGGGCGACCCTTTTGAAAACCTTTTCTCCAATGGAACCATCCTGTATGAATGAGAGTCTTTAAAATAATGGAAAGTCTGTCTAGAGAGTTATTG

12001 TTTGTCCATCAAAATATGCATAATAGTCATTTAAATCCCACTCTAAGCTTGGTAATATTACCTTTGACATACTGTATTTCTGAAACCTCAACCAAACAAG

12101 TTTTTTTTTTAAAGCAAAAAGAAAGGAACATTTTGCTTTATTATTACCAGTTTACAATTAAAGAAACAGCTAATGTAAGTTGTAAACGAACAAGCATTGA

12201 TAATTAAAAGTATTTGAGTGTTTTTTAAGAAATGTGGCATGTCACCATAGATTCTATGAATACAGACGCTTCATTATGGAGTGAAGAGTCCTAGAGCATC

12301 ACTGACCTCTATGTTTTAAACTGTGGAGGTTAAAACAAATAGGGGTTGTCTTGATTTGATGAAACACTTTTTTTTACAGTTAAAGTCGAGCTGATTATCT

12401 CATATCTAAAATAATCAAATAATTGTTTAAATAAATTTTGTTTAAAAAGGAATAAATATTATTTTCAGGGTAAATGAGCTGTGCTTAAAAAATTATCATT

12501 AAATTAAACAATCTAATAGTTGTTTTTATACCTCCTTCAGTGTTCTTGCATCTTTAAAAAAATAATTTTAAGCATTGCAATAGCTGCAAATACTGAGTTT

12601 TGGTTAACTGCAACATGGGATGGCTGGAATTTTGTACTTTCATTAAGTGCTTTTTTTTCTTGTATAGCAAAGATAGCCAACATAAAACAAGAAAAATAAA

12701 AACAGAGAACAAAGGTTTAATCATATTTATCAAATAATAATTTTGTTTATTTTAATGATCTGATTGTTTTATATATGCCGATTGACAAGCACAAATCATA

12801 TTTCGCATTTAAAAGTATAGTCATAGGTCAACGCAATTCCCGATACAATGCAAATAACTAGAGAATCACAAAAAGTGTGATGGGATTTATTGACCAGCTC

12901 AAGTAGTTTCTAGATTACTCAAAGCATGGAAAAAATAATTTTTCCAAAAAGATATAATTCCTAATTCCCATATTCTCATACTTTTTCCTATATCATCATT

13001 GCCATTTTGGCAATTCAGCACATTCATTTAAAGGTTAAAGAGCCACCTATCCCTAAAATGACCCCAATAATTGCTAACAAGGTTCATCATATGAAGAAAA

13101 TGAGACTGAAAGCTGTTGACAAAATACTTAAAATGCAGCCACAGATTTTCTGGACAGATCCTGACATGAAGGTGCTTGAGACAACCCAAGTTAATCACCC

13201 TGTTAAAATAAGTAGACATGTTGAGTTGAAAATACTCTTCTTTTTTTAATCTTATATTTGTTAAAACACAGTCATACATGAGTAAAACATGAGTAAAAGT

13301 AATGACATCAAACATTTGTAGAGACATCCCAGTCAACTCTTAAACATTTTTTAAAAGAAAATGAGAGTTGCTGCGACAACTTAGCATCACCTCTCGACAA

13401 ATATTTGTCATTTTTTTCACCGTCTGAAAAGTTATTCTAAATCAGTAAGTTGGAGTCCATTATTTTTAGTGTTTTCAAACAAAAGTTACAACTTGACAAC

13501 TTCGCCACCAATTGAGTAGCAGTAATTTCCGATTGTTTTCGAACGCACCACAACCGATCTCTGATTGGACGACACAGGACGAAAAGGCCCTTCCCCCTTC

13601 GCTTGGAAATCCCCAAACAACGTGTTCGACGCTGCAGTCGCCGATTCAAAATATTTTGGCTTTTGGCTTTTTTGAAAACGTCTTTCCAGCCTCTTGGATA

13701 ATAAATAGCGTATTGTTGTCGGTGCGATTCCCGACCATCAAAGGTACAAACTATTATCTTATAAAACATGTCGAGAACTTTGAAGAACAGCATATTTACA

13801 GCGAACAAAGTTTAGCTAGCTACCGTTAGTAAAAACGTCGTTGGAACGTCGTTAGCTAGCTAGCAGTAAGCTAATTCCAAGTTTTCCTGCTAACGATAAA

13901 CCACGGTTTATTTGTTTGTTTATTAGCTTTGTATCGAATTTCGTTCTTGTAAACATTTGAATCAAAACTAGTTTGGTAAATGTTTATTTATTTTACACGT

14001 TAGGATGACAGAAAAAAATCACGCTAACTTCTTAATTCTTACAGGAAGTTAGGCCTCTTGAGCGGAAGATGTTTGTTTTGATTTTGTACAGGGCATTATT

14101 GGGGGCATTAATAGAGGGCTTTTAACATGCTATTGCAAGAATGATGCGCATATTTGAATAAATATAAAATGGAACTATTTAAAAATATTTTGTGTCGAAA

14201 GCTTTAACTTGGATCTGTAAGGTATTTGCGTTAAGCTTCTGGGATTTATCAAAACACTGATTTGCTTCAGAGAAACTCGATTACTTTTTATTAAAGTCAG

14301 GTGTGTTTTTGAGCACAGGGAAGAAAGTGAAACCAGCGCATAATTGGTGGGCTGGAGCTGAGTAAATTTGGACGAAACCATTACAAATGAGGGGGTAAAG

14401 GGCCCGTGCAGATAACTGTTGTTTTATATTATATTGCATTTAAAAAGGGTATTTTAAGCATAGTAGTAGTTTCCCTATGCTTCAAATTGCATTTAAATCT

14501 TGCTTTTGCCTTACATTGATTTCATTCCTAAAGCTTCTGCTTTCTCCCCCCTCATTTGATTCTGCCTCTCCTTTTCTGAGTTGGGAAAGC**ATG**GATCCTG

14601 TACCCGACCTGCCCGAGAGCCAAGGTTCTTTTCAAGAACTCTGGGAGACTGTGTAAGTTACTTTTAAGACTTCCCCCACATATTTTGGGCACAAAAATGC

14701 ATAAGGGTGTATATTAACTAATCGTATTTTAGTAGTTATCCTCCGTTGGAAACTTTATCACTTCCAACTGTAAATGAGCCCACCGGGTCATGGGTCGCAA

14801 CGGGGGATATGTTTCTCTTGGTAAGTAGGAAAGTTCAAGCCATAAACTACATTTTTATTTTTGTCATTGGACTTTCGCTACCCAATACATTGTGCATGTA

14901 CTCACGGTTGGAAGAGCCTTGGTGCAAAACGTCCAACTGGTGCAAATCTCTTGAATCTTTTTTCCAGTATTCCCACATTTGAAAGATTTGGTGTCATA**GA**

15001 **ATTC**CGGCGGGGCACAAATTGTAATTATTAATTCCTAATCTGTTTCAAATGTTTGGCACTTCAATGAATTAAAAAAGAAACCAGACGGCACTGGACAAAG

15101 CAAAAGTCCCTGATTGGTCAAAGTTTGACCTTGTTTCACTTAATCTCCCATTTAATGGATGCGCATTTTAGTACGTAGAGCCCCAAAACGGTTAGAGAAA

15201 CTTCCGTAGCAATGTTTTGAGCACACGTGCCCAAAATGTGCCTAGACTCCCGTTCACATGGACTTCTCATTGGTAGTGGTAACTTGAACGCATGGTGTGA

15301 CTGTAGTTTCACTGATTTGTAGCAATGGGAATTGCAGGAGGAAAAAAATAATTGCCAGTATGTGGCTTTTGATTCATCTCCAAACATTTTTAGTTTTCTC

15401 ACCCTGTTTTTGTAAAATGCTGACAGGATCAAGACCTCAGTGGAACCTTCGATGACAAGATCTTCGACATACCGATTGAGCCAGTTCCCACCAACGAAGT

15501 AAACCCGCCACCTACCACCGTCCCGGTGACCACCGACTACCCGGGAAGCTACGAACTGGAACTTCGTTTTCAAAAATCTGGCACTGCAAAGTCTGTGACG

15601 TCCACAGTAAGTTGTGAAAATCACTTTTTTTCCCATTTGTAGCTTTCCCCCAAAATTAGTTTTCTTGTATATCCATCTTCGTGTTATTTAATATTACTAA

15701 TGCTGAAACATTTCTTTCCTTGGAAATTTGCATCCAGTCTATATTGTTTGCTGATATAAAATGACTGTTTTGCAACCTGACATGATAAAGACCTTTGTGA

15801 GAGGAAAATGATCAAACTGGTTCATCGTACGTTAGACTCACTGCATTAAAACCCTTTAAGTCTTTATTTTATTATTTGGTCATTTATACAGACTAGTGAT

15901 TTATTCCGTTGTTTCTAAATATATTAAATGAAGTCTTACCTCGCGGCAGCGTTCTCATTCATTCATTCTCTCGTAGCATTCTTCTTAGGGTCATTCAGAT

16001 GATTGAACGCTGAATTTCTAGCTCTGCCCACGCTCAAATAGCCAATGGTATTACCGTTTCCACAGTGACTCGACGCTCTGAGTCATATGGTTGGCCGTAT

16101 AAGTTAAGGTTAGGGTGGACTGTGCTGAAACGGCCCGCCTCCTGCGCCTGGTGCTTGTCTGCGTGGGGAGAGGAACGGCCTTCTGGTGAAGCACAGCTCT

16201 GACATGACGGGACATGTTCCTACACTCACTTCCTTTGGGCGAGAAGAGGTTGATGAAAAGGAGAAGGTTACAACTTTAATTTATAACTTGTTATAGAAAA

16301 TATAAGGAGGACAAAGATTTTAAAGTGGACTTAATAGAAAGTTTAAGTGATTTTAAGTTTGTAGTCTTATTTTTATTGGTTGCTTTTGTTTTTATGTTTT

16401 CACTCTGAGGTGAATATGTCAATCACAGCTGCGGTAAGTTCATTTTTTTTTTTGGTTTTCTGTGTTTTCTTATTCCCAGTACTCTGAAACTTTAAATAAG

16501 CTGTACTGCCAGCTTGCAAAAACCAGCCCCATAGAAGTCCGGGTAAGCAAGGAGCCTCCAAAGGGCGCCATTCTCAGGGCCACTGCGGTCTACAAGAAGA

16601 CGGAGCACGTGGCGGACGTGGTGAGGAGGTGTCCCCACCACCAAAACGAGGACTGTAAGGAAAACAGACAAATTCTGCAAAGAGAGGATTTGATATTTAT

16701 CTGATAAACAGACTGGAAGTCTTAGTTTGTTCTTTCTTTCCCTTTTCTCCATCGACTGTTACATTTTATAGCTGTGGAGCACCGAAGCCATCTGATTCGG

16801 GTGGAGGGCAGCCAGCTGGCCCAGTATTTTGAAGACCCTTATACCAAAAGGCAGAGTGTTACAGTTCCTTATGAGCCCCCGCAGGTAAAGCTGCCCCAGT

16901 CTGGAATTGTCTTTGCTCTTAAGCTAACGAATGGCCTGATTTTTCACCTCAGCCGGGCTCTGAGATGACCACCATCTTGCTTAGCTACATGTGTAACAGC

17001 TCGTGCATGGGGGGGATGAACCGCAGACCCATCCTCACCATCCTCACTCTGGAAACCGAGTAAGTTTAGTCCCACTTTGCTCATCCTTTGATCCATTTTA

17101 AAAGCGTTTAAAGTGTTTTTTAACTATGGTTGTGCTGTTTTCAGCCAAAATAAAGATGTATTCAGACTGGGAAAACCTCTGCATTTAGACTGAGGAACCT

17201 CAGAGCGAAGTGATGCTCAGTCCCATTGGAAACGAACCAAGACCTCCTCCAAAGTTGGGTCCGAGAGCGGTTCCTGGTACTGGATCAGGGTCCGCTCGAG

17301 TGTATTCAGACTGAAAATTTGTTCTGGATTATCAGGGGAAACAAACTCTGGTTCATTTAAAGTGAACCAGATGTGTCCAGTCTGAATACACCCTAAAGAA

17401 AAAAAACTTTCATCTTCTTGGCCCCTTGCTGCAGAGCAGCAGGATTTCCGTTGGCGCGGAGAAACCCCGCCCCCTTGTCTGCTCCCTTATCCTGGGAGCA

17501 GGGAGTATATTTTCTTTTACTAATGAACATTTTCCATCTGTATTTCTTCATCTGCCGATTCACAATTTGAAAAAAAGAGATTCTCAGAAATGCATTTTTG

17601 AGTTTAATTTTCTCTATATGCATCCTCCATCGCAAGAAAAGAAATGCCACAGGAACATGTTAACACTATTTTTATCCGAGTGGGTCTCTAAAGTAGAGCA

17701 GGCTGCAGTTTAGTAAAGATACATATTCCTAAATGAATTCTGTGGTTTCAGGTGTTCATGTTTTTGCCTGTTCAGAAGTCGTGTTAATTTTTGGTTCTTC

17801 TTTTCCCAGATCAACCTTGAAAAGTCTCCACCTCTGCTCTTATCAATCTCTTGCAGGGGTCTGGTTCTTGGCCGGAGGTGTTTTGAGGTCAGAATCTGTG

17901 CTTGCCCGGGGCGGGACCGCAAAACGGAGGAGGAAAGCCGGCAGAAAACGCAGCCCAAAAAAAGGAGTATGTTGTGTGTCCTTTTTAGTTTGTAAATGCC

18001 GTTGTGTGAGGTTTTTCTTTGCTAAGTTGTTTTCTTCACAGAGGTGACACCCAACACCTCCTCCTCTAAGAGGAAGAAGTCCCACTCAAGTGGAGAGGAG

18101 GAGGACAACAGAGAAGTCTTTCATTTTGAGGTAAATGAAAAAGGAAAATGCGGCTGCGTAGAAATGATTTCTGATTTAATGACAGATTTCTGATGTAGGT

18201 TTATGGGAGGGAGCGCTACGAGTTCCTTAAGAAGATAAACGATGGCCTTGAACTGCTGGAAAAGGAGAGGTAAGGAGTCTGGGGGCAAATTAAAGGAGAG

18301 TTTTTATTTTAAAATATTCGTAATTGTATAATTATTATTCATTATTTTACTTTAATTATTTTATTTCCCAACTAATGACTCATCCGCGATGAAAAAATAA

18401 TGTCCAGTATAAACTGAGGGAAGTGACTTAATATATAAACAACAAACAAAAATAGAAAAGAAGCAGTCCGCTCCTGCACTTGATTAGTCAAGTGAACCGA

18501 GCGACCGTGTCTCTGAGCAGAGCAGTCGGACTTATAGTTTTGTGTTTGAGGGGAAGGGAGGATGCTTTGTTCCGTGTGCGGGAGACTTCAGACTGCGATC

18601 TGTCCCGCAGCTCTAGGTGAACGAGCAGCCAAATTCAGGAGAACCATGTCTCCTGTCTCCGGGCAGAGCTCGGACCGTCAGCTCACACGGCGGCTTTGGG

18701 ACAGTGTTTGAGCTGTTCAAAGCAGAAATTCCGCTCAGTCCTTAGAAACCGTTCAAACAATCACGTGAATTTGTGTCCTGACAAAATAAAGGCTGATGTT

18801 AGTCTCACATATTTACGTTCAGCCAAGATGCAGATTGCAGCATTTCCCGCACTGATTTTATGTTCATCAAAGGCTAAATGTTGTTGTTAGCGCGTGTTAG

18901 CCTTCCAACCCTGGCTTCTTCTATGGAGGCTCAAACTGTTCATAATTAAATAAATAAATACGACCGTGTGCAAATACACAATAAACCAATAAATCACTCA

19001 TAAATAAATAAAAAGATCGTGAAATTGTGAAAAACATGCACACAGATTTTAAAATAGCCATTATATTATACAATTATTTTCATTTTGCTTTTAAAACAGC

19101 ATTTTTAAAATATTAATTTTAATCATGTTGAATATTATTGTTATTCTATGGCTTTTTTTCAGAACTTCGGATTTCAAAATCAATATTTTCCAAAGTAGCT

19201 TTGTTTTTATTTCACATTGCTGTTTTTCAGAATGACATTTATTTCATGAAGAGCTTTTCCAGCATAATGAGTCTGTCTGTCAATCAGTGAAAGTGGGTGG

19301 GATCTAAACGCCCATTGGCTGATCTCTGGAAATCTACTCGTATTCCTGGAATCCCGCTCTGTGGTGTGCCACTCAGAGACATATTTCTGCGACATCCGCA

19401 CGGCTTTGCTGACATTGACTGATCAAATGGAGACGATCTGTCTGCTGCAGAAGATGCGAACATCAACGACTCTGTTTTTCTAGTTTGAGGGTTCGTGGGG

19501 ACCACAGAGGAGCTTTCTTTAAGTGCCATTCATCCACTTCTTGAAGTTCCCGTTGAAATACTCCCGACTCTGTGTCCCAGTTTTCCACCCCAAAAGTCAT

19601 CTGAAAACCGCCAGACCCAACAAAAAAACGCCCGATCCTTATGGCCGTGGACCATGGGGGTCATAAATGAAAATGCAAAATGTCTTTTTTGCATTGGAAA

19701 ATGCAAGGGGTTTATCCTAAAATCCTCACACTATTCTCTCAGAGCTTTTCTCTGTAGCTGACACTGCTCCGCCGGCGTCCGTGCTCACAGATCCAGTCTG

19801 TCGTCACATCTGTTCGCCTGACACGCCATCACCCTTGCGGCTCCCGGGAGGCGAGCCAGCCCGCTCGGGACTCCTGAACCTTATGATATCTTTTTATTTT

19901 CATGTAGACAATATTTATAAATCAGGTCCCCTGGTTTTATTTTTCCCTCTCGGGTTAACGCAGGACGGCAAACTTTCAAACTCGGTCAGAGAGAGATGGA

20001 GGAAAAACCGCTGAAGTGGCCGTCATGAAGGGAACTGAAGCTGGGATGATCTCATGAACTGAAACGTGGAAACAGGATTACCACGTAGAAGTCTCAACAT

20101 TCTCCGTGTCTACCATGCATTGTAAACGAGACAAACACCATGTAGCGACCAGGCTAAAAACATTAGCTGGTAGCTCCTCCAACAGGCGGCCTGGTGCGTT

20201 CACTGAGCTCCAGTACATAGAAGCTTCGCTGAACTCCAACAGCCTCCCAGCCTAACTTAGACCACTATTATATTTTCATGAGAAAGAAGTAAATGCAGAC

20301 CCTAAAACCACCCAAAGCCATTTTTACCTGCAAATGTAGAACCAAAACCGCCAGACCTGGCAACACTGTGTTGAGGTGCATACTCCCTCTAGTGTTGAGG

20401 GTGTGCATTACGCCGTCACGTTTGCTGAAGCAGCTTTGATCGATGTAGTTAGTTAGGGTGCTGCTGCTTATGTCTGAGTACAGGAGTAGCCATTCACAAA

20501 AGCGTCTCCGCCTTGTCTGTTTGACAGTTTTCTGCTGGAAAAGCTCTTCATGAAATAAATGTCATTCTGAAAAACAGTAACATGAAATAAAAACAAAGCT

20601 ACTTTGGAAAATATTGATTTTGAAATCTGAAGTTCTGAAAAAAAATTCATAGAATTATAATAATACTCAACATGATTAAAATTAATATTTTAAAAAGGCT

20701 GTTTTAAAATAGTGAACAGTTTTTTTAAGCAAAATGAAACATATTTTATAATAAAATGGCTAATTTAAAATCTGTGTGCATGTTTTTCACAATTTCATGA

20801 TTTATTTATTTATTTATGAGAGATTTATTGGTTGATTGTGAGATTGCACAAGGTCATATTTATTTATTTAATTATGACCAGTTTAGGACTCCATAACTTC

20901 TCGCTCCTTTGTTCCACAAAAAAAGCACTGACCACACGGATTAAAAACAAAAACAAAGAACAGGGCGCTTCATAATAACCACAACAATAATAAAAGCACG

21001 TTTAGAAGATCATCTGGTATATTACCGAGCTGCCAGATATCTATGTATGTGGCAGCTTGGCTTATACAGCGGAACTCAGTTCCTCTTCTGGTATTTCAAA

21101 CACTACAGCACATGTGCGAATGAACCCCGAAAGTGAGACCCATGTGAACGTTTGCTATAATCGGAAAACATTTTTCTGGGCCCATGTACACGGTTGAATT

21201 CTAATCCGACCATTGATCCCATCAATATATTTTGCACATGTAACCGCGGCTTATGGTGTTGATTCTCAGCGTGGCGGGGGCGTGGCATCACGATGGGGTC

21301 TGTCCATCGTGATGCCAGTCAGCCATTACGACGAATAATGATATCGTCCATCAGCACAACCCTACCGCCAACCCCGAAAGGGATATAGAGTCAAGAAGAT

21401 GGATGGAGAAACCGCCCTTCAACAGCCCAGAGTTTCTCATGTTTAAGAATCTGTAGTTCTGTTAGCAAATGTTTTTTTTCTCTTGGACTAGCTCCAAACA

21501 TTAGCCTACGATGTTTATGCATTTCCATGAAAATTCCAATACACTGTCTATCAACGGTACTTTTCGTGTGCAGAAAAACATTCAGACAATTGTCATCAGA

21601 AAAAAGCTAGACGTTTGTAAACCAGTCATGTTGGAGTGGCATGTTGTGGGTTTTTTGTGACACGGAAGTCATTTGAATATACGTTCATCCACCCACGGCG

21701 TAACGTTGACACATGCGGGAGTTGTTGCTCTGCTTATTATGTGATGCTCTGTGTGTAGCAGGAAATTTCATCAGCTCAGTAACATGTTTTCTTTATTGTA

21801 TCCCCCCCCCCCAGCAAAAGTAAAAACAAAGATTCTGGGATGGTCCCCTCCAGTGGGAAGAAGCTGAAGAGCAATTAAAGAACTCATGCATGGAACAATT

21901 TGATTTTTCTTCTTCTACTTCTTGGTTTCATAAATTACTTTTTTACATTTTTTAAAATTTGTACTCTTCTTTGTAACCTATAGTTAATTGGCATGATCAG

22001 GATATTTGTAGGGGGGGGTCGGCTGGATCACCCCCCCAAAACGGCGTTTTTTGTTTTTGTACAAGATTTATTGGGATAAAGGTAATTTGTCCTCAGTATA

22101 ACAAATAATGAGTAGGGGATAAAACTTNNNNNNNNNNGTTTTGTTTTGAGAATAATAGTGCAGCACTTTGGTTTAATGGACCAATGTTTTAGTTCGACAC

22201 TGACCACACATTTAGAGGCATTTTTGTCTTCCAGTATGCTTATATTTTATTATAATCTTAGTTCTGTATGAGTTTCACACTTATATATATATATATATAT

22301 ATATGTGTATATTTTTTGTCATTTGTAATGATTTACTTTTTTTTTGTACCAAATTCCTTGTTAAATAAAGTTTTTCTGCTCCAGAGTTTCCATGTCTTTG

22401 TAAACATTTTAAACATTACAAGAAAACAAAAAATACAAAACTGGAACAACATAAACTAGGTGGGAATTTTAGCAACGTTTAAAATATATATATATATTAA

22501 AAAGGCATCATATTCAAATTGATAACTTTGGAAATTATCAAAAAGTTTGAGTATTCCTTTTTTTTAACTTGTCCAACAGCTGAGCAAATAAGAGCTGAAA

22601 GCCTCTTGTGTTGGACATATTTTACTGTTTCAACATGGCGTTATGAATCTTCTGACAAGCTGTATATTTGAATAAACCCTTTTTGTAATTTAGGCTAAAC

22701 TTTAGATTATATTTGTAACTTTTATTGTTGGACCGGACTGAAAAGTTTTTCGTCAAAATTGACTATTTCTTTTAATAAACCAGACACAGTGGCTAACTGT

22801 GTTAAACTGGACCTTTTGTGAATTTGGAAAAAAAGAAACCAAAAGAACAATCCCCCATTCTAGTGAGTCTGTGGATTATTGTGCGTGCGTCGGCTCAGAG

22901 CAATGTGTTTTGCATGTCCGGTTTTGTGGGTTTAAAAATGCCTGCATTTGTACGTTTTTTTTCAAATTGAAAGGAGCTCTTTAGCTTTAGGCAAATTTGC

23001 TGTCATTATGGTCGTGGTTTGTAATTCCTCCCCCGTCCGTCAGCACGATCCAGGATCTTGGAGGATTGAAAAACCAAGCGACTGATGAGTCTGCATTTAT

23101 TTAGCCTGGAGACTGCAGGTGTCGTTTGTGAGTTACTACAGATTTTTGTCTCTGTAGTTCTATCATAGCTTTCCTGTATGCTTGTGGGAATACTTGCTGC

23201 CCAAAATGGGAACGCTACTTTTGGACAAAGCCTGTATTTTCATTTGTTTGGGTGAGTTTTTAATTAATTTATTCATTTCTAAATCTTCCAGTCTGGTTAA

23301 ACTCATGTGTTAATTGGCTTCAGGCAATATTAGGTTGTTTAATAAATGATTAAGTGTTTAAAATGATTTTTACCAAGAATAAATTTGTAAATCAGACGAT

23401 TAAAGACTTGTCCCTCTTGACTTTATATTCATTTACATGAAATTTTCTACACATTTTACATTTTTCTTTAATTTATACTACAGGACATATAGGTCAGGAT

23501 ATATTTTCTGGAAAGTAAAAGAATAATACTTTGGGATATTTTCATGCCAAACACATGCTGTGGAGCTCGTATATAATAACTGCTAAGCCTGTTTTTGCAA

23601 CGTTAATGAGGTCAGTGTTGTGTACAAATGTTTAAAAAAAATGAGCTCCTTTAGTTTCCATATCTATCTGAAATGTTCCATGTAAACAAATATGTTCATT

23701 TTAGATAAAGATAACAAATGAATGCAAAACTGTATTATTCTTACTAATATTTATTAATGGAAAGAACACATTTGAATCTTTGTAATTATTTGTGGAAACT

23801 TAATTTAATGTTGGTTTGTTTTTTATCCCTGGAGGCAAAAACTATTCAAATGTTTTGGTAAACTGTTTTTTAAATGAGTCAGTCATCAGTCATGTCAATA

23901 GGGTCTAAAGTAAGTCCAGTTCATTATCAGGTTTATCTGTTGGCGTGTATTGAGATCTGAGCAAAGTGCGGTTCTAGGACCCGTTTCAGAAACCCCCTTT

24001 TCTTCCCCCATCCTGTCCTATGATGCTATGAGCGTTTTCTTTTAGAAAGTAAACCAGCTTTTTGATGATCACCAATGAATTGGGAATTAAATATAAAGAA

24101 ATATACATGCAATATAAACTCAATAATTCATTTCCAATTAAAGTAAATTGTGTTAAGGCTTTAGTAATTCCACATTGTAAATTGCACTTTTAAAGAAAAT

24201 TAATTAAACTTGGTTTCTTAATATAATGATTTGAAAATGCTGTTGAATCAGCAAGCTAATTAATTTTCATAAATCTAGTTTTTGAAAGAAAACTAAATTA

24301 TGAATTATGAAATTCTCTACGTCTGTGTGACCTTTTCGTCTAAGGAGGCACAGTTTTTTGGCATTTTTAACGTTCTGATGCTAACATAGACTAATTTCAT

24401 TCGTCTTTGTTGTTTTATGATGAAACGAGACGTTTCATCTGGAGGAGGGGGGCGTATCTAACACTGTTTTCATTTGGTGTTTTCCTTTGGTGTTTCTTGT

24501 GCGCATGACAGGTTGATGATGTAAGGTGACAAAAGGACTTCTGGGTATGTGAGTGAAAAGGAAAATAAAGTTGTGGTCCTCTACACCTCTGAGCTCCTTA

24601 TTTTACATAAGACACTCCGCTTTACTGACACAAACCCCGGAAAGGCAGCTACACTAACAAGAATCTGATTTTGAGTTGCCAAAAGGGTCAGATTTGTTTT

24701 TGTTTTTGAAACCTACCCGGAAATAAAACTTCACAAAATGCTCCACATCTTTTATCTTTAAGCACGGCTTCAATATACAGACAACTTTGGTGTTTTTCTT

24801 GTAAATTAATGACTTTTTTCTCCTAAATGTACAACTTTTTCTAGTAAATTCAAGATTAAAGTCGTATATTTATGACTTAAGTCATATATTTATCACTTTA

24901 AAGTCGTGTATTTATGGCTTAAGTCGTATATTTATGACTTTAAAGTCGTAAAGTCGTATATTTACGACTTAAGTCGTATATTTATGACTTTAAAGTCGTA

25001 AAGTCGTATATTTATGACTTAAGTCATAGATTTATGACTTTAAAATCATATATTTATGACTTAAGTCGTATATTTATGACTTTAAAGTTGTAAATTTACA

25101 ACTTTGTTCTTGTAAATTTACAACTTTATTCTCGTGATTTAACAGTAATTCCTTTTTTTCTTGTAAATGTATGACTTTAAAGTCATAATTAAAAAAAAAA

25201 AGTAAACTGGCCCTAAAACTCCTTCGTATTTATCAAACAAACACATGATAAAGGAAACGTTAAAAAGAGGTGAGGGTAGCAGAGGACGGGGGACTGACTG

25301 TTTATCCAGCAGAGGTGGCTGTTGAATCAGGAAAGCATGAGGACGTACACAGACAACTCTGGCAGGAATCAGTTCAGAGACAGAGCAAAATCCAGCTTTT

25401 ATTTTGATAGTCCACTTCCGCTAATTGGCAAGTGCATTTGCATGTTAGCTAAATAAAGTTTGGACCTAAATATTCATCTACAAAACAAAAAATGTACTTT

25501 AAAGCTAAATATTTAATTTGAACTGAAATATTTAATCAACAGATAAATATTAAGTCATGAAACTTTAATGTTTTGTTTAGAAAATAAATATTTAGGTCAG

25601 ATCTAAATATTTAATTAGCAAAATAAATGTTTAATTTACAAACTGAACAACTAAACTAAACATTTAGTTTGGAATCTAATAAAATATAATTATAAAAAAT

25701 ATTAAACTTTAAATAAAAAATAAGCTTACTAACTAAATATTTAGTAAGTAAATTCGACATTCATAAATACATGAATCAAAAGATGCAAATTTGTAAAAGG

25801 AAACCTGTTTTTCCTACAAAAACATGCAAAAACCCCAAAAAATATTGCTGTTTAAAAATTTGACTGTGCAACTTTACAACAGCACCCCATACTGAAATCA

25901 TGCAAATCTTAGATTTGATTAGAAAACTGTGTTCACTTTACAGATAAAACGGAAAATGCTTTTTCTGTTTTCATAGTATGTCTCTCTCGTCGTGTTTTTA

26001 AACTCACTTTCCAATTTGCTAACTTTACATTTAGCTGTCGTGTGACACACTTTGACTGTATATAAGAACTGGACTGAGCGCGTGTGACGTCACCCATGGA

26101 AAATGGTTTACCTCCGACTCCAACAAAATGTCGTCAATTCAGTCGCCATTTGTTAAGATACAGACTCCACCATGTTGGAGACAGACAATGTCGTTAGGGA

26201 TTGATCCAAGTCGGTGTGAGTCATTGTTTCTATGGAAACCACTCTTCCCAATCACTGATGATCTTGTTTGAAGTCCACACCTTTACCACTTGAAAGTGGG

26301 CTACAAGGAATCTGTCAATCAAAGATTTTAACTCTTCACGTAAGATCACATTGTTTTATTGAAGCATCTGATTAAGCAGTTTATAACTTAAATAACTTGC

26401 AATAAAGACAAAAATAATTAAAAAAAATTAGCATCAAGAACGTGTTTAAAAAAACTAAGAATGTCATTTTGATCAATAGAATGACTGACTGAGTAATAAC

26501 TGTTTATTTCTCTATAGGGAGGAGCCACTCAGTCCAGTTCTCATAAACAGTCAACCACAATCATATGGAAGAAATGAACAAATTAGAAATGAATGTTGTG

26601 GTGGTTGAAGTCTGAATACTGAACATCTACTTTCCCTCTTTTTTTTTAGACTTTTGCAAAAGGTCAAAAGAATTTCAACCAGACAAATTCAAAAAAGAGA

26701 TAAAAACCGGAAAAGGGTGTTTTAAAAAATCTGAAACATGTTCATTTACAAACGTACTTGTTTCTTTCTTTTTTTTTTAGCTTTGATTCTCACATTCACA

26801 AGTGCTGAAAGACAGTACTTCAACGAGTCCCAACAGCTTACCTGGGATGAGGCCAGGAGCTACTGCCAGGTACTTTTATGTTTAACTCACTTTCATGAGA

26901 ACTTTACTCACAATTTTACATTTCTTATGAGTACTTTTGCTACAACAGATTTGGTTATTATACAATAAAGCAAAGCTGTAACATGATTGTAATTATGTTC

27001 ATGTTTCTTGCTGGTTCTTCTGTTACTTGTGGCTCCGCCCTGTTAAAGCAATTTATCTGCCTCCGTTAAATCTCAAACTCTTTATTGTCCTCTCTCACAT

27101 TGATTAAGTTCATCATCACTTTCACTAAACCTACAGATTTCATCTCTTGTCATCTTTATGTTTCTTCTCCAGAAGCCTCGACCTGAGCATCTTTTTACCC

27201 TCCTGAACACGTCTCTTCCTTTCAGTTCCACTTTCCTCGCTTCTTTTAAAGTCCCACTCCGATCACCTCATGGTCTGTTGTAGTGTTCCCAGAGGTGTTT

27301 TTATTATTATTATGCAGTTTTTAGCCAAAATAAAAAAAAAACCTGTTATTTTGCAGGACATAGTTTCTGCAGAGCAAGTTGTGGGCGGGACTGTCGGTAT

27401 GGAGTTAGCCCCCTCCCCCCTCCCCTCCATTGTTGAGAGCTCTCTGTTCACACGCTATCCTGCTAGTTTCAAGAGTACCTGGCTTCAAACATTAACGTCC

27501 AGAACCAGAATTCCTGAACCGGAACTTGAAGCCCACATTGCTGCTTTTATTTTGAAACAAAGACGTTCAGGGATCTATTTGTCTGCTACCGAGTGGAGCG

27601 GAGCAGGCAGCTTGTGGCCCACTGACTGCAGCACCTACATCACAGCAACTGGCTTTCTCAAACTGCATTTTTTCGTCTGTTTTTAATAAATACTCAGGAT

27701 TGCAATTTTATGCATAGTTTTGTTTATATATGACCTCCATCTTCAGAAAAAGGCCACAAGAACATGTTAAAAAACCTAATTTAAATCTTTAAGTCTATTG

27801 GTCCTATTTGTTAATCTTACCCAAATTATTACACTCAGAAAGAAGCAGTGTATTTTTCTCCAGACCGTCCATCAGCAACAAACACATCATCGGCGGTCTA

27901 ATCACCGTCTTGCATACCTTTTCTTTCAGTCTTACTAACGCTCATTTCCACCTGTTTCAACCTGACCGAATCGGTTATTTGTCATAGCAGCTTTGGACAG

28001 GTGGTTCTAATTGAGGAAAGTCCTCCACCTTCCTCACCAGCTGTTCCAGGTGAACCTCTCCTTTCCATTGTGTTGTTTTCACTTAAGCAACTCCTTTCCA

28101 AACCTTCTGCTTGATATTTGACACCACAGCTCCTCCCGACCTTCTTTGTTCTTCTCTAGAAGGATCCTCAAAGCAAATATTGCAGCTGTGGTTCCTTTTC

28201 TTTGGATCAAACTGTACTGTTGCTCAACAAATGCTCATTTCTGACCTCAGCCGAGGTTCCTCGACTCTTTCCCTTAACTTAATAGTGTGACTCATCAGCT

28301 TTTTTCCTCTGGAGTTTCCATAACTCTCCCTTGTCCTTAATAAAGATGGGCACCAGTCCACCTATTTTCTCCAAAATATCGCCAAATCTGCTGCCACCTC

28401 TCCCGAACATTTCCATATGTCCAAAAGCAAATCCTCCGCACTAACCGCCTTTCCAAGCCTCTTCCTCTTCAACACTCTGCAATTCTTCCTTATTGATAGT

28501 CAACGTTTCCTGCTCCACAAGCGTCACCTCTTTGCTCTCTAACATTTCTCTCTGTCATCTACTCCCCATGATTTGTGATGCACTTGTGGAACAAGTCAAC

28601 AGATTTTCAGCTGTATCCTACCTCACCTGTTACACACCCCTCCATCGTTGTCTCTGCCTCAGCAACCTGTGCAAATTCATCTTTCCTTCTTCAATGTTCA

28701 ACCTATCGTGAAATAATCGACTCAGAAAATTCTCCCTTTTTATAAACTTCATTCTGTTTCCAACCAGGCCTTTTTACTCATTATTCTGTGCCTAACCTTT

28801 TTCTTTTTAAATAGGCTTTTTAACTTCCTTGTTCCTTTTTCGTCTTTGCATATGACACACAAAGTATTCTCCAACATGTTGGTGTCTATTCTATTAGTCT

28901 ACAAGCACCTCTCCACTATCTATCTTTTATCTACTATCTTTTTTCAACTCTTTCCTGAAAGACCAAATAAATATATTCTTCACAGCCTGTATGTTGTGTG

29001 GTCACACTCTCCCCTGACCCCACTTTGGACTCACTGATCTCCTAGACGACAAAAACCACACAAAACTTTTGTTCTAAATCCTTCTATGCTCTTGTGTCTT

29101 GTCCAACCTAAATCCGGCATTGTCAATTATTATGCAATACTCTCCTGAAAACCAAAATAATAAATCACTTTATCTCCATCTCTGTTTCCCTCACAAACAT

29201 GTCTCCACATTCCAATCCCAAATTTGATGGCAACCAGGGAATACAAAATCTTGCAGTTTTTCAAATGACCACCAGAGGGAGACTGCTTTCAGTCTTTAGA

29301 CTGAAAAGCACCATTCACTTCTGTGTTCAAAAGTCACAAGGTTTTGAAACCGAAGAGAATCACATACAGCCTGGTTTTAGAATCTTAACCTAGGAATTAT

29401 TGACCTATTTAACCCTACATAACCATCTTTTTCATATTTAACGCACACATGTATGAGGTCCCCATCTCAATAGCGTGATCAAAACATTGTATTTAACTTG

29501 TTTCATGTTTTCTGCCCTGTAATCGAAACTTAGAGCTTGAACTTGTTCGAGAATTTAAATATCTATAAAAAAAATCATCTTAATTCAAAAGACTAATTTT

29601 CTGTCAATGTTTTGAGTAGTGGGCTTTACAGGGTTAAAATCCAGTATGTATATGTCTAATGTAAACATGATCACCAATTTTTTTACTTCTGGAAATATTT

29701 TGCATTATCTCATGCCCTTTTCATGAATTTCACATTCTGCTATTACTTTTATTCTACATGTTGAGCATAACCACTGACAGATTTCACCATTAAATCCTAC

29801 TGTTTCTTGGAGGATCTAGAAAGACCCAATACAATCAAAATCTTGTTTTTGATGTTTTTAACATATTCTTGTATGTCTTGTTTTTGTTATATGTCTTAAA

29901 TTTTTTGGGACAATGCCTCTGCCATTTTTATAGCATTTTTCTGATGATGGCAGACATATGTTTAACAAAATTAAGCTCAAAATTGCATTTCTGAGTATTT

30001 T

**Experimental design for Southern (EcoRI-5’external probe)**

1 kb

**external probe**

E

**A**

**wt *p53***

**null *p53***

E*

**E**

E

14,605 bp

E

**E**

E

13,340 bp

**9,253 bp**

7,988 bp

*neo*

**neo probe**

E, *Eco*R1

(A) The external and neomycin probes were labeled with digoxygenin and used for detection of HR events and insertion of targeting vector, respectively. These two probes are specific for the endogenous *p53* locus (outside the short HR arm) and *neomycin* gene in the targeting vector, respectively. In the wild type *p53* allele, the external probe is hybridized with an 8 kb *Eco*R1 digested fragment. After replacement of exon 2 to 7 by targeting vector (the backbone and *tk* got lost after HR), one *Eco*R1 site (asterisk) is missing and this fragment is converted into one of 9.2 kb.
